# Bilateral and symmetric glycinergic and glutamatergic projections from the LSO to the IC in the CBA/CaH mouse

**DOI:** 10.3389/fncir.2024.1430598

**Published:** 2024-08-09

**Authors:** Isabella R. Williams, David K. Ryugo

**Affiliations:** ^1^Garvan Institute of Medical Research, Darlinghurst, NSW, Australia; ^2^School of Medical Sciences, University of New South Wales, Kensington, NSW, Australia; ^3^Department of Otolaryngology, Head, Neck and Skull Base Surgery, St. Vincent’s Hospital, Darlinghurst, NSW, Australia

**Keywords:** binaural hearing, sound localization, lateral superior olive, glycine, glutamate, principal cells

## Abstract

Auditory space has been conceptualized as a matrix of systematically arranged combinations of binaural disparity cues that arise in the superior olivary complex (SOC). The computational code for interaural time and intensity differences utilizes excitatory and inhibitory projections that converge in the inferior colliculus (IC). The challenge is to determine the neural circuits underlying this convergence and to model how the binaural cues encode location. It has been shown that midbrain neurons are largely excited by sound from the contralateral ear and inhibited by sound leading at the ipsilateral ear. In this context, ascending projections from the lateral superior olive (LSO) to the IC have been reported to be ipsilaterally glycinergic and contralaterally glutamatergic. This study used CBA/CaH mice (3–6 months old) and applied unilateral retrograde tracing techniques into the IC in conjunction with immunocytochemical methods with glycine and glutamate transporters (GlyT2 and vGLUT2, respectively) to analyze the projection patterns from the LSO to the IC. Glycinergic and glutamatergic neurons were spatially intermixed within the LSO, and both types projected to the IC. For GlyT2 and vGLUT2 neurons, the average percentage of ipsilaterally and contralaterally projecting cells was similar (ANOVA, *p = 0.48*). A roughly equal number of GlyT2 and vGLUT2 neurons did not project to the IC. The somatic size and shape of these neurons match the descriptions of LSO principal cells. A minor but distinct population of small (< 40 μm^2^) neurons that labeled for GlyT2 did not project to the IC; these cells emerge as candidates for inhibitory local circuit neurons. Our findings indicate a symmetric and bilateral projection of glycine and glutamate neurons from the LSO to the IC. The differences between our results and those from previous studies suggest that species and habitat differences have a significant role in mechanisms of binaural processing and highlight the importance of research methods and comparative neuroscience. These data will be important for modeling how excitatory and inhibitory systems converge to create auditory space in the CBA/CaH mouse.

## 1 Introduction

The auditory system is constantly tracking inputs received from the two ears. Principal neurons of the lateral superior olive (LSO) combine excitatory glutamatergic projections from the ipsilateral cochlear nucleus (Cant and Casseday, 1986; Doucet and Ryugo, 2003) with inhibitory glycinergic input from the homolateral medial nucleus of the trapezoid body (MNTB) that is driven by activation of the contralateral cochlear nucleus (Kuwabara and Zook, 1991; Banks and Smith, 1992). The convergence of this binaural information using interaural level and timing differences is sent to the inferior colliculus (IC) (Grothe and Park, 1995; Franken et al., 2018; Williams et al., 2022). Electrophysiological recordings from the inferior colliculus reflect the output of the LSO with maximal excitation leading from the contralateral ear and inhibition when the sound is delivered to the ipsilateral ear (Hind et al., 1963; Kuwada et al., 1984; Grothe and Park, 1995; Park et al., 2004; Ono and Ito, 2015; Ono et al., 2020). The computation of sound location is achieved by the manner in which the auditory system utilizes neural responses created by ongoing interaural differences in time, level, and spectral cues (Mosieff and Konishi, 1981; Tollin and Yin, 2005; Yin et al., 2019). These binaural functions are crucial for the brain to sort the various auditory streams that bombard the system constantly and to make sense of our auditory scene.

The lateral superior olive (LSO) contains a heterogenous population of neurons with either ascending or descending projections, which are suggested to function separately in information processing (Ryugo et al., 2011; Williams et al., 2022). Defining its ascending circuitry is considered crucial to understanding mechanisms of auditory stream segregation. It had been suggested using tract-tracing methods that principal neurons of the LSO have bilateral and symmetric projections to the IC (cat. Adams, 1979; greater horseshoe bat, Schweizer, 1981; gerbil, Nordeen et al., 1983; mustache bat, Ross et al., 1988; Mexican free-tailed bat, Grothe, 1994; Wistar albino rat, Kelly et al., 1998); we quantitatively support this conclusion for the CBA/CaH mouse (Williams et al., 2022). There are, however, disagreements as to the symmetry and chemical nature of these projections: (1) ipsilateral projections are entirely glycinergic and inhibitory (Willard and Martin, 1984; Saint Marie et al., 1989; Moore et al., 1995; Mellott et al., 2021), (2) low frequency neurons project ipsilaterally, whereas high frequency neurons project contralaterally (Glendenning and Mastereton, 1983; Oliver, 2000), and (3) low frequencies project contralaterally and high frequencies project ipsilaterally (Henkel and Brunso-Bechtold, 1993).

The data that include transmitter chemistry with the corresponding laterality of LSO projections to the IC are also conflicting. Published reports to date suggest that ipsilateral projections are primarily glycinergic, and the contralateral projections are glutamatergic. These differences, however, could be due to variations across species (cats, Saint Marie et al., 1989) or chinchillas and guinea pigs, Saint Marie and Baker, 1990; bat, Klug et al., 1995; Long-Evans rats and Swiss Webster mice, Ito and Oliver, 2010; gerbils, Mellott et al., 2021; CBA/CaH mouse, Williams et al., 2022), age (Helfert et al., 1989; Nerlich et al., 2017), cell staining methods for determining amino acid chemistry including in situ hybridization (Mellott et al., 2021); immunohistochemistry (Storm-Mathisen et al., 1983; Wenthold et al., 1986; Koch and Sanes, 1998; Williams et al., 2022); and pathway tracing such as HRP histochemistry (Glendenning et al., 1992), dextran amines (Williams et al., 2022), and selective uptake and transport of radiolabeled glycine (Saint Marie and Baker, 1990; Glendenning et al., 1992). In the context of differences in the species, age of the subjects at the time of examination, and methods employed, variations in the results should not be surprising. The challenge is to advance our knowledge about binaural hearing by understanding the brain differences as they relate to species, species habitat, and methods of research.

This present study sought to confirm the bilateral and symmetrical LSO projections to the IC (Williams et al., 2022) and to extend our understanding of excitatory and inhibitory effects in the mouse. Using retrograde labeling and antibody staining methods in the CBA/CaH mouse, we sought the following: (1) to determine the projection pattern of glycinergic and glutamatergic LSO neurons to the IC, (2) to assess somatic size of the different classes of LSO neurons, and (3) to infer LSO influences on sound localization mechanisms.

## 2 Materials and methods

### 2.1 Mouse model of hearing

This study was conducted in line with the Australian Code for the Care and Use of Animals for Scientific Purposes (2013). Usage of all animals were in accord to the Animal Ethics Committee protocols (Animal Research Authority: 20-02 and 21-13) and utilizing the principals of Replacement, Reduction and Refinement with the approval from the Garvan Institute of Medical Research Animal Ethics Committee. A total of 20 CBA/CaH mice of either sex and aged between 4 and 6 months old were used. CBA/CaJ mice (Strain #000654) were imported from The Jackson Laboratory (Bar Harbor, ME) by the Australian BioResources Facility (Mossvale, New South Wales, AUS), renamed CBA/CaH as requested by The Jackson Laboratory, and an inbred colony established. These mice were chosen because they exhibit exhibits stable auditory brainstem response (ABR) thresholds for up to one year (Zheng et al., 1999; Sergeyenko et al., 2013; Muniak et al., 2018) and are commonly used to model normal animal hearing (Berlin, 1963; Ohlemiller et al., 2016).

### 2.2 Hearing status

All animals underwent ABR testing prior to experimentation. Mice were anesthetized using ketamine/xylazine (100 mg/kg; 20 mg/kg), and placed in a double-walled, sound-attenuating chamber (Sonora Technology, Gotenba, Japan) on a heating pad. Once areflexic to a toe-pinch, the recording, reference, and ground electrodes were placed in the skin on the cranial vertex, left pinna, and biceps femoris, respectively. A MF-1 speaker (Tucker-Davis Technologies, TDT) was positioned 45^°^ off the midline and 10 cm from the pinna where alternating condensation and rarefaction click stimuli (100 μsec square wave pulses) and tone stimuli at 4, 8, 16, 24, 32, 40, and 48 kHz (5 ms duration, 0.5 ms rise/fall) were generated using a software-controlled signal processor, RZ6/BioSigRZ (TDT), and delivered from 90 to 30 dB SPL in 10 dB decremental steps to either ear separately. Stimulus presentations (*n* = 512) were delivered at a rate of 10/s for each level and the evoked responses were amplified (RA16PA/RA4LI; TDT), bandpass filtered from 0.5 to 3 kHz, recorded, and averaged (RZ6; TDT). Hearing threshold was defined as the sound level at which the peak ABR amplitude was four times the standard deviation of the average baseline noise level (Bogaerts et al., 2009). Only mice with normal auditory brainstem response thresholds and audiograms (Zheng et al., 1999; Taberner and Liberman, 2005; Muniak et al., 2018) were used in this study.

### 2.3 Tract tracing from the inferior colliculus to the lateral superior olive

Following ABR testing, each individual animal was placed in an atraumatic DKI stereotaxic head holder. The surgical approach to the IC began by making a skin incision on the dorsal surface of the head to expose the cranial sutures, bregma, and lambda. Approximately 5.2 mm posterior to bregma, a unilateral craniotomy (roughly 2 mm^2^) was made overlying the IC using a #11 scalpel and a surgical pick. Pressure injections (0.5 μl at a rate of 100 nl per minute) of the retrograde tracer, Fluorogold (FG; 4% in saline, Fluorochrome, Denver, CO, USA) were made using a manual microinjector (Sutter Instruments, Novato, CA) with the needle tip directed into the central nucleus of the IC at a depth of 1.0– 1.5 mm by a micro manipulator (DKI Model 961, Tujunga, CA) using the stereotaxic coordinates of Paxinos and Franklin (2008). Following the IC injection, bone wax was applied to cover the craniotomy, and VetBond tissue adhesive was used to close the incision site for the post-surgical survival period. Retrograde tracer was placed in only one ICs in order to distinguish LSO neurons with ipsilateral or contralateral ascending projections (Supplementary Figure 1).

### 2.4 Tissue preparation

Fourteen days after an IC injection, animals were administered an intraperitoneal, lethal injection of Lethabarb (200 mg/kg bodyweight). When the animal was unresponsive to a paw pinch, the chest cavity was surgically opened and the heart isolated. The descending aorta was clamped, the right atrium punctured, and an 20g surgical needle, connected to a feeding syringe by flexible tubing, inserted into the left ventricle. The upper body and head were perfused transcardially with 3–5 ml of 1% sodium nitrate in phosphate-buffered saline, followed by 60 ml of 4% paraformaldehyde (in 0.1M phosphate buffer, pH 7.4) delivered at a rate of approximately 20 ml per minute. The head was removed, the calvaria partially opened to expose the brain, and the head post-fixed for another 2–3 h. The brain was then completely dissected out of the skull under an operating microscope and the brain post-fixed overnight at room temperature in 0.1M buffered 4% paraformaldehyde. The following day, the brain was embedded in a gelatin-albumin mixture hardened with 4% paraformaldehyde, sectioned into 60 μm-thick sections using a vibrating microtome (Leica VT1200S, Nussloch, DE), and collected in serial order in buffer using 24-well tissue culture plates.

### 2.5 Immunostaining with either GlyT2 or vGLUT2

FG-labeling of the LSO principal cells was observed following unilateral FG injections into one IC. Sections containing the SOC were counterstained for the glycine transporter 2 (GlyT2) using rabbit anti-GlyT2 (*n* = 7, Cat# PA5-69264, Thermofisher, Massachusetts, USA) or for the vesicular glutamate transporter 2 (vGLUT2) using rabbit anti-vGLUT2 (*n* = 5, Cat# 42-7800, Thermofisher, Massachusetts, USA). Sections were incubated in 0.1% Photoflo (Kodak, Rochester, NY, USA) for one hour, followed by an hour in 20% normal goat serum. Sections were washed three times in buffer and incubated at 4°C overnight in either 1:1000 rabbit anti-GlyT2 primary antibody and 2% normal goat serum or in 1:1000 rabbit anti-vGLUT2 primary antibody and 2% normal goat serum. One section per case was not exposed to the primary antibody and used as a negative control.

The following day, sections were exposed to either rabbit anti-GlyT2 or rabbit anti-vGLUT2 antibodies, rinsed in buffer, and placed in 1:200 goat anti-rabbit IgG cross-absorbed secondary antibody, Alexa FluorTM 488 (Cat# A-11008, RRID:AB_143165, Thermofisher, Massachusetts, USA). After one hour, sections were rinsed in buffer, mounted on standard microscope slides, and coverslipped with VectaShield (H-1400; Vector Labs, California, USA).

The principal neurons labeled from FG injections were viewed under the fluorescent microscope with a wide band ultraviolet excitation filter (Zeiss 19012 AT Filter). GlyT2 or vGLUT2 was viewed under the fluorescent microscope using 499 nm excitation filter [Zeiss (Colibri) Filter Set 59 HE]. The specific fluorescent label from the IC injections and antibody staining prevents cross-over of the label when viewing through the microscope. The MNTB served as a positive control for the GlyT2 neuronal labelling (Supplementary Figure 2).

### 2.6 Cresyl violet Nissl stain

Cresyl violet (CV) staining was performed on separate cases or sections whose fluorescent signals had faded using a protocol modified from Humason (1979). This basophilic dye stains acidic components of Nissl bodies, ribosomes, and chromatin to reveal the cell bodies and nuclei of neurons (and supporting cells and vasculature). The sections were hydrated in distilled water for 5 min, followed by a 10-min incubation in 0.1% CV dye at room temperature. The slides were rinsed in distilled water, followed by rinses in 70% alcohol, 95% alcohol and then differentiated (95% alcohol with 10 drops of glacial acetic acid) for one minute to remove excess staining. Rehydration in decreasing concentration of alcohol (one-minute periods in 70, 50, 30%, and distilled water) further removes excess CV for air-drying overnight and cover slipping with Permount the next day.

### 2.7 Quantification of LSO neurons and microscopy

Examination of tissue was conducted using a Zeiss AxioPlan microscope fitted for brightfield and fluorescent microscopy. The following objectives were used with our Zeiss AxioPlan microscope: 100x Oil Plan Neuofluar, NA 1.3; 40x Plan Apochromat NA 1.2; 25x Plan Neofluar NA 0.60; and 10x Planachromat NA 0.25). The high numerical aperture (NA) of each objective optimized final image resolution (300 dpi) and avoided empty magnification. Serial sections of the CV-stained LSO were imaged from the rostral to caudal extremities of the nucleus, guided by the facial and pontine nuclei respectively, to determine the boundaries of the LSO. Criteria for neuron identification and counting were established to reveal three cytologic categories: large periolivary (PO) cells, medium-sized principal cells, and small cells (Supplementary Figure 3).

Further analyses of neuron types were made using projection data and transmitter histochemistry for all sections through each LSO. Cell counts were performed in the contralateral and ipsilateral LSO nucleus for principal projecting (FG) neurons, GlyT2+ only neurons, vGLUT2+ only neurons, and those that double-labeled (FG and GlyT2 neurons or FG and vGLUT2 neurons). Brightfield photomontages (40x objective) at 3 focal planes through each section containing the LSO were compiled and stacked (300 dpi resolution, Adobe Photoshop 2024). Without moving the x-y position of the microscope stage, z-stacks of fluorescent photomicrographs through the same LSO were collected using the UV excitation filter, and the GlyT2 and vGLUT2 neurons from images taken with the 499 nm filter. Manual counts were conducted for the principal cells and for cells labeled with the antibodies; counts for double labeled neurons were made by superimposing the micrographs from the two different filters to determine which cells were double-labeled.

Neuronal criteria were established for the counts and included only cells with a clear, sharp somatic outline and a visible nucleus (Supplementary Figure 4 and Supplementary Table 1). Other blurry globules, holes, and artifacts in the tissue were ruled out using the criteria for labeled cells. A ratio of all the principal IC projecting neurons labeled in the ipsilateral and contralateral LSO was calculated for all cases. Photomicrographs (40x objective) of neurons labeled with either GlyT2 or vGLUT2 were imported into *Photoshop* and the cell body outline was drawn and filled on a separate layer to represent the cell body silhouette area. TIFF files of the drawn silhouette area were loaded into FIJI ImageJ2 (V 2.14.0/1.54f) to quantify the area of the GlyT2 and vGLUT2 somata.

Counts for the projecting neurons, glycinergic neurons, glutamatergic neurons, and CV-stained neurons were compared and related to previous counts reported in the literature. No correction factor was applied in these counts (Hedreen, 1998). Statistical analyses were performed on the data output from the neuronal counts, ratios, and cell size using Descriptive Statistics, Mann Whitney two-tailed test, and Two-way ANOVA using Šídák’s Multiple Comparison Test (Prism 9, 2021 GraphPad software, San Diego, CA USA). Means, standard deviations, p-values, and statistical tests are provided.

## 3 Results

Principal neurons with ascending projections and intrinsic neurons with descending projections exhibited similar somatic anatomy using standard tracing techniques, but could be differentiated by chemical stains: intrinsic efferent neurons stained with cholinergic markers, whereas principal neurons did not. Within this grouping, there are principal cells with ipsilateral or contralateral projections that have been inferred to be glycinergic or glutamatergic, respectively (Glendenning and Mastereton, 1983; Saint Marie et al., 1989; Saint Marie and Baker, 1990; Moore et al., 1995; Mellott et al., 2021). In most studies, glycinergic neurons are reported to project almost entirely to the ipsilateral IC (Saint Marie and Baker, 1990). In the gerbil, 76% of the principal neurons are glutamatergic with contralateral projections to the IC (Mellott et al., 2021), whereas in the C57BL/6 mouse, 98.6% of vGLUT2 neurons projected to the contralateral IC (Haragopal et al., 2023). In the CBA/CaH mouse, half of the retrogradely labeled cells had projections to the ipsilateral IC and the other half to the contralateral IC (Williams et al., 2022). Our goal was to determine the chemistry associated with the laterality of these IC projections. We labeled glycinergic and glutamatergic neurons using antibodies directed against the transporters, GlyT2 and vGLUT2, respectively, in tissue that contained retrogradely labeled LSO principal cells following retrograde tracer injections into the IC.

### 3.1 Labeling of LSO principal cells co-labeled with GlyT2

**FIGURE 1:**
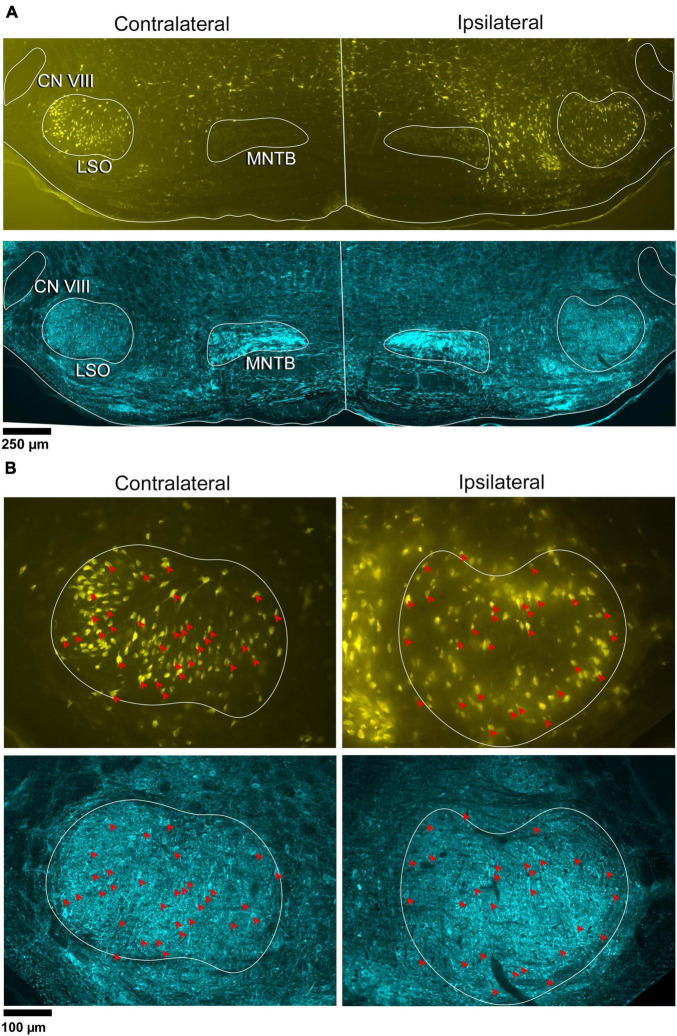
Photomicrographs of IC projecting neurons (yellow) and GlyT2 neurons (blue) in the LSO. Principal neurons were labeled via a unilateral injection of FluoroGold (FG) into the right IC. This tissue was then counterstained by GlyT2 immunohistochemistry. **(A)** Low magnification montages of the SOC (10x objective) show the contralateral and ipsilateral LSO with retrogradely labeled principal neurons (top panels, yellow) and GlyT2 staining (bottom panels, blue). Micrographs of the same LSO were captured using a different fluorescent filter to reveal both types of labeled neurons. The MNTB contains well-labeled GlyT2 neurons as a positive control. **(B)** Higher magnification images (25x objective) of the same LSOs shown in A, illustrating double-labeled FG and GlyT2 neurons (red arrowheads) in relatively equal numbers. These results also show that some GlyT2 neurons project to the ipsilateral IC, some to the contralateral IC, and some to neither. FG, FluoroGold; GlyT2, glycine transporter 2; LSO, lateral superior olive; CN VIII, vestibulocochlear nerve; IC, inferior colliculus; MNTB, medial nucleus of the trapezoid body; SOC, superior olivary complex. Scale bar equals 250 μm **(A)** and 100 μm **(B)**.

Large unilateral retrograde tracer (FG) injections were made into the CNIC to label LSO principal neurons. A bilateral, mostly homogeneous labeling pattern of neurons filled both LSO nuclei (Figure 1, yellow). Within the LSO, a few larger, polygonal neurons were somewhat concentrated around the dorsal hilus within the borders of the LSO. These multipolar cells were distinctly larger than principal cells and could also be found lightly scattered throughout the LSO. They resembled the previously described periolivary neurons (PO) located within and around the LSO (Williams et al., 2022).

In the same tissue, GlyT2 immunohistochemistry was performed to reveal labeled cells and fibers (Figure 1A-row 2 and Figure 1B-row 4). The majority of GlyT2 labeled neurons were medium in size, fusiform in shape, and resembled principal cells (Williams et al., 2022). These cells were distributed throughout the LSO. A subset of GlyT2-labeled neurons was small and oval-shaped, featuring scant cytoplasmic staining by CV; these did not co-label with the retrograde tracer injected in the IC. Another small population of neurons had larger, oblong somata residing within and around the LSO, and resembled previously described PO cells (Supplementary Figure 5A).

**FIGURE 2:**
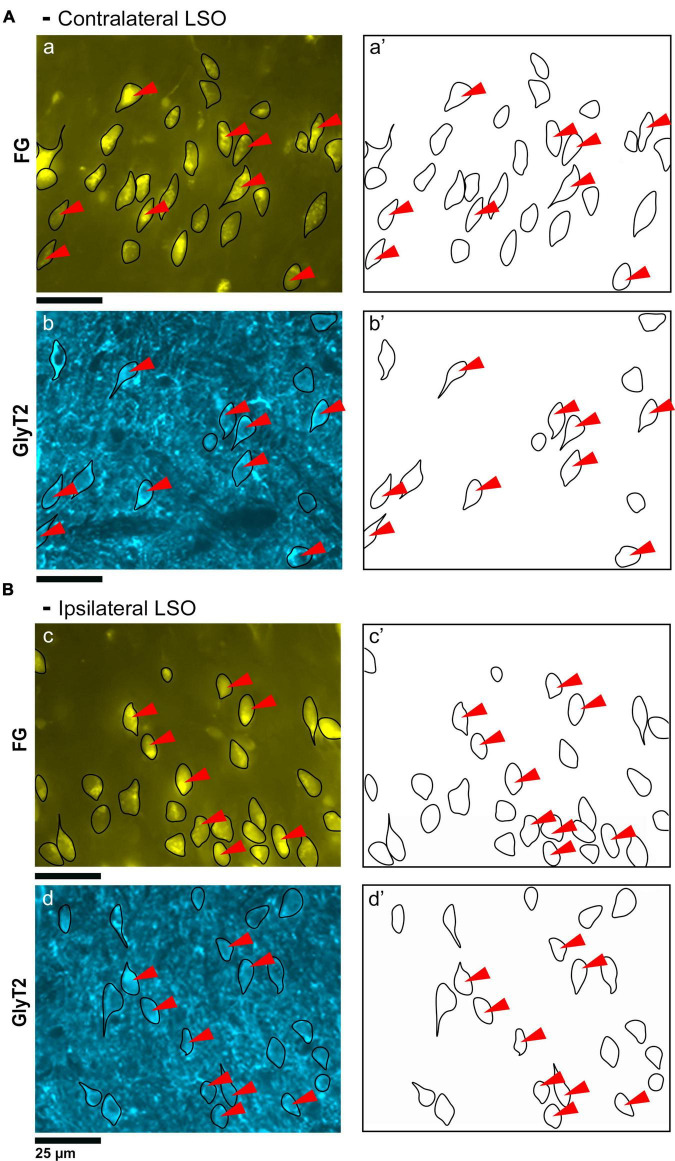
Photomicrographs (40x objective) and drawings of LSO principal neurons (yellow) labeled via FG injections into the IC and counterstained with GlyT2 (blue). **(A)** Top row shows FG-labeled neurons (a, yellow with black outlines) and corresponding drawing of the labeled cells (a’) from the contralateral LSO. In row 2, GlyT2-labeled cells are shown with black outlines (b) and corresponding drawings (b’). The red arrowheads indicate the double-labeled cells in the photomicrographs and drawings. **(B)** Upper row shows ipsilateral projecting neurons (FG, yellow) with black outlines (c) and corresponding drawings of the labeled cells (c’). The bottom row shows that the ipsilateral projecting GlyT2-labeled neurons (d, d’) have a similar size and shape compared to the contralateral-projecting neurons. The IC-projecting cells that co- label with GlyT2 immunostaining are indicated by red arrowheads. FG, FluoroGold; GlyT2, glycine transporter 2; LSO, lateral superior olive; CN VIII, vestibulocochlear nerve; IC, inferior colliculus; MNTB, medial nucleus of the trapezoid body; SOC, superior olivary complex. Scale bar equals 25 μm.

**FIGURE 3:**
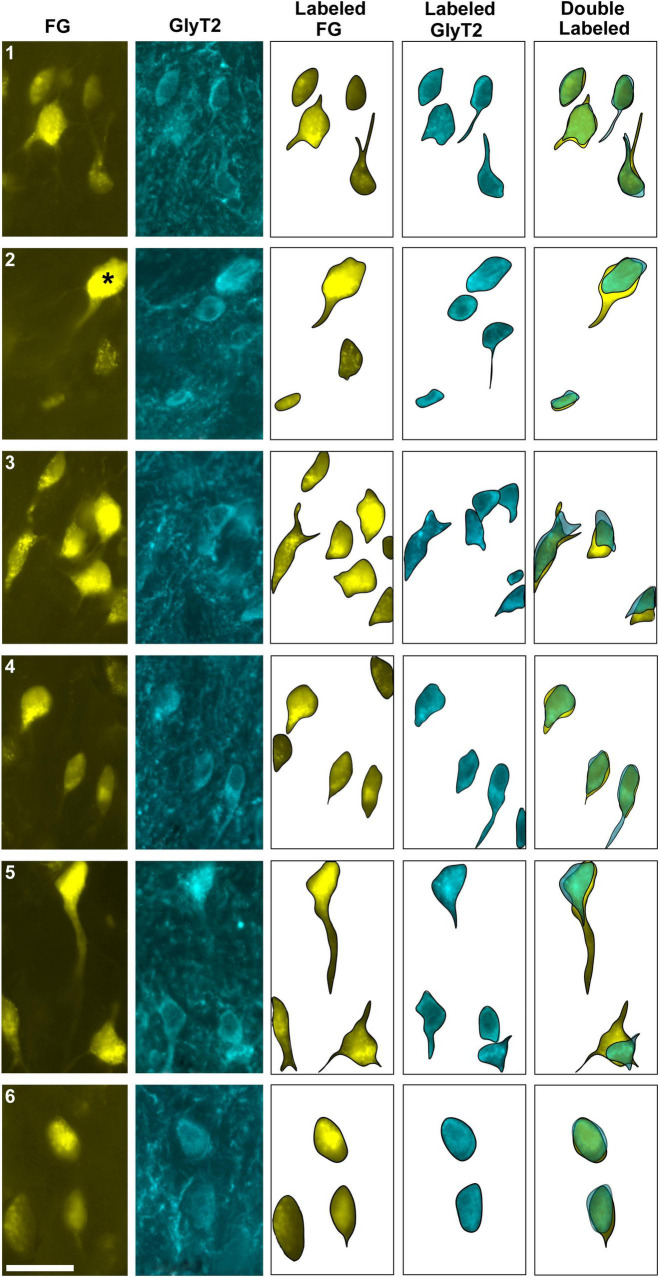
Photomicrographs (100x oil objective) and drawings of double-labeled LSO neurons (rows 1–6). Principal neurons were first labeled via a unilateral injection of the retrograde tracer (FG) into the IC (column 1). This tissue was then stained by GlyT2 antibodies (column 2) revealing that some FG neurons were also labeled for GlyT2 in either the ipsilateral or contralateral LSO. Not all principal neurons were double labeled. The neuronal shape of FG and GlyT2 neurons from either the ipsilateral or contralateral LSO are shown (columns 3–4). Double-labeled neurons containing FG and GlyT2 are identified by their overlapping position and near identical somatic features (column 5). An occasional large neuron resembling periolivary neurons (* in row 2) was doubled labeled. Not all GlyT2-stained principal neurons project to the IC, and not all IC-projecting neurons co-label with GlyT2. FG, FluoroGold; GlyT2, glycine transporter 2; LSO, lateral superior olive; CN VIII, vestibulocochlear nerve; IC, inferior colliculus; MNTB, medial nucleus of the trapezoid body; SOC, superior olivary complex. Scale bar equals 25 μm.

Principal cells projecting to the IC were co-labeled by GlyT2 in the ipsilateral and contralateral LSO (Figure 2), were surrounded by neighboring GlyT2 positive axons and dendrites, and featured an opaque nucleus that was readily identifiable. The nuclei in FG-labeled neurons were obscured by the cytoplasmic fluorescence. By illuminating and photographing the fluorescence of the retrograde tracer using one fluorescent filter, and capturing the fluorescence of the GlyT2 label with another filter, the two images of the same section were overlayed to reveal a population retrogradely labeled cells that co-labeled with GlyT2 (Figure 3). Notably, not all labeled IC-projecting neurons were GlyT2-positive, and not all GlyT2-positive neurons were IC-projecting. Unlike previous reports (Glendenning and Mastereton, 1983; Saint Marie et al., 1989; Saint Marie and Baker, 1990; Mellott et al., 2021; Haragopal et al., 2023), the co-labeled neurons in our study demonstrated that glycinergic neurons projected in equal numbers to the ipsilateral and contralateral IC.

### 3.2 Labeling of LSO principal cells co-labeled with vGLUT2

**FIGURE 4:**
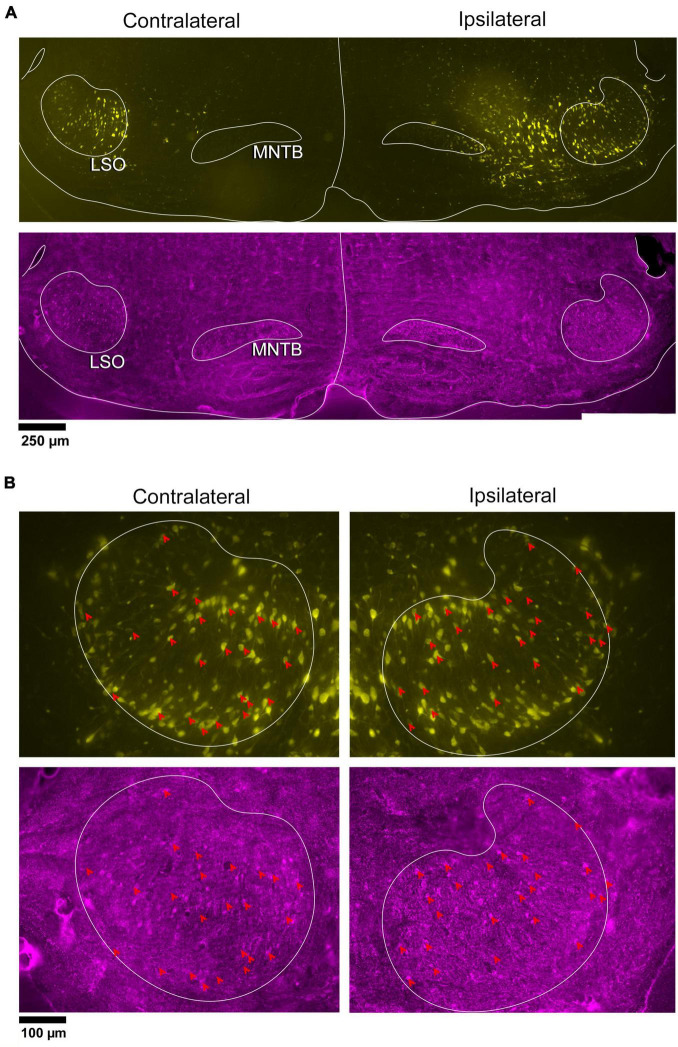
Photomicrographs of IC projecting neurons (yellow) and vGLUT2 neurons in the LSO. Principal neurons were labeled via a unilateral injection of the retrograde tracer, FG (yellow), into the right IC and counterstained with vGLUT2 immunohistochemistry (magenta fluorescence). **(A)** Low magnification montages of the SOC (10x objective) show the contralateral and ipsilateral LSO with retrogradely labeled principal neurons (top panels, yellow) and vGLUT2 staining (bottom panels, magenta). **(B)** Higher magnification images (25x objective) of the same tissue in A, revealed double-labeled neurons for FG (upper panels) and vGLUT2 (bottom panels) in the contralateral and ipsilateral LSO (red arrowheads) in approximately equal numbers. Some vGLUT2 neurons project to the ipsilateral IC, some to the contralateral IC, and some to neither. vGLUT2, vesicular glutamate transporter 2; others as in Figure 1.

**FIGURE 5:**
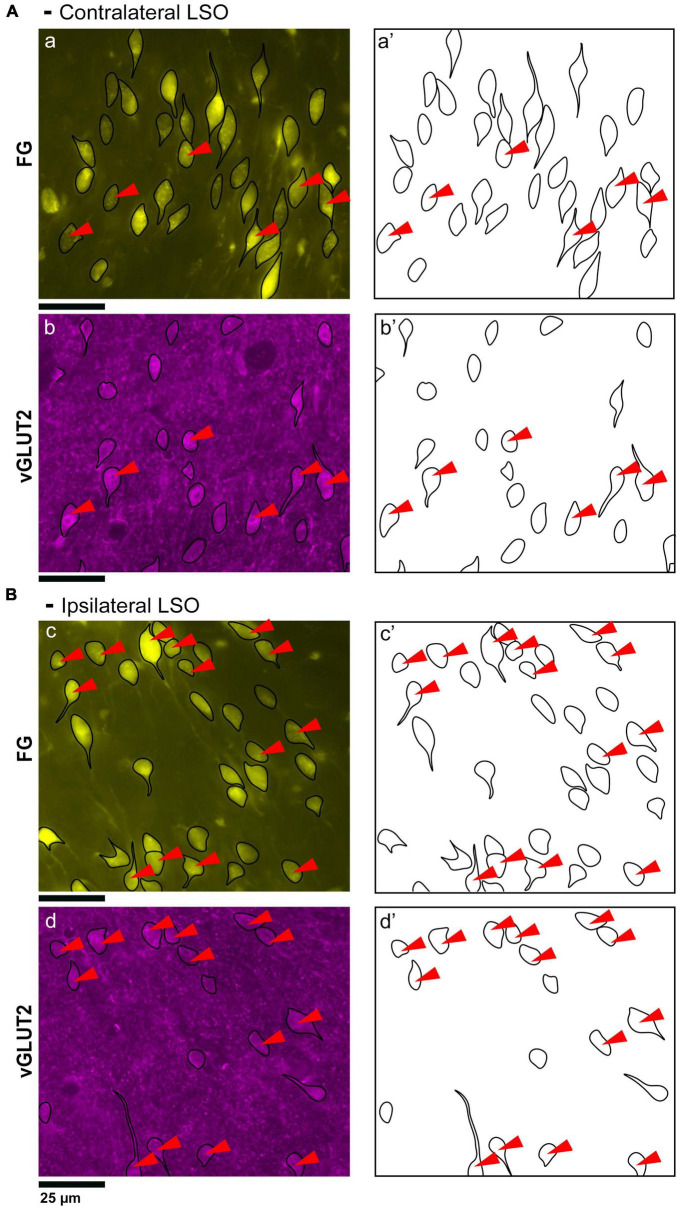
Photomicrographs (40x objective) and drawings of LSO principal neurons (yellow) labeled by FG injections into the IC and immunostained for vGLUT2 (magenta). **(A)** Top row shows FG-labeled neurons (a, yellow with black outlines) and corresponding drawing of the labeled cells (a’) from the contralateral LSO. In row 2, vGLUT2-labeled cells are shown (b, with black outlines) and drawings only (b’). The red arrowheads indicate the double-labeled cells in the photomicrographs (a, b) and schematic drawings (a’, b’). **(B)** Row c shows ipsilateral projecting neurons (FG, yellow with black outlines) and corresponding drawings of the labeled cells (c’). The bottom row shows that the ipsilateral vGLUT2-labeled neurons (d) can be matched to the contralateral-projecting neurons by location and somatic shape (d’). The double-labeled neurons are indicated by red arrowheads. These results confirm that some vGLUT2 neurons project to the ipsilateral IC, some to the contralateral IC, and some not to either. FG, FluoroGold; GlyT2, glycine transporter 2; LSO, lateral superior olive; CN VIII, vestibulocochlear nerve; IC, inferior colliculus; MNTB, medial nucleus of the trapezoid body; SOC, superior olivary complex. Scale bars equal 25 μm.

In a separate set of animals, vGLUT2 immunohistochemistry was used to counterstain the retrogradely labeled IC-projecting neurons in order to compare and contrast co-labeled neurons within the ipsilateral and contralateral LSO. The labeling pattern of vGLUT2 for retrogradely labeled principal neurons was similar to that of GlyT2 labeling: there was a population of ipsilateral and contralateral IC-projecting neurons that co-labeled for vGLUT2. Not all IC-projecting neurons labeled with vGLUT2, and not all vGLUT2-labeled neurons were IC-projecting (Figure 4). The vGLUT2 antibodies labeled cell bodies and surrounding fibers, which created a level of background staining. Regardless, the fusiform appearance of principal neurons was evident (Figures 4, 5). Consistent with other staining techniques in the LSO, a small population of distinctly larger, multipolar neurons tended to reside around the dorsal hilus and resembledPO neurons (Supplementary Figure 5B).

**FIGURE 6:**
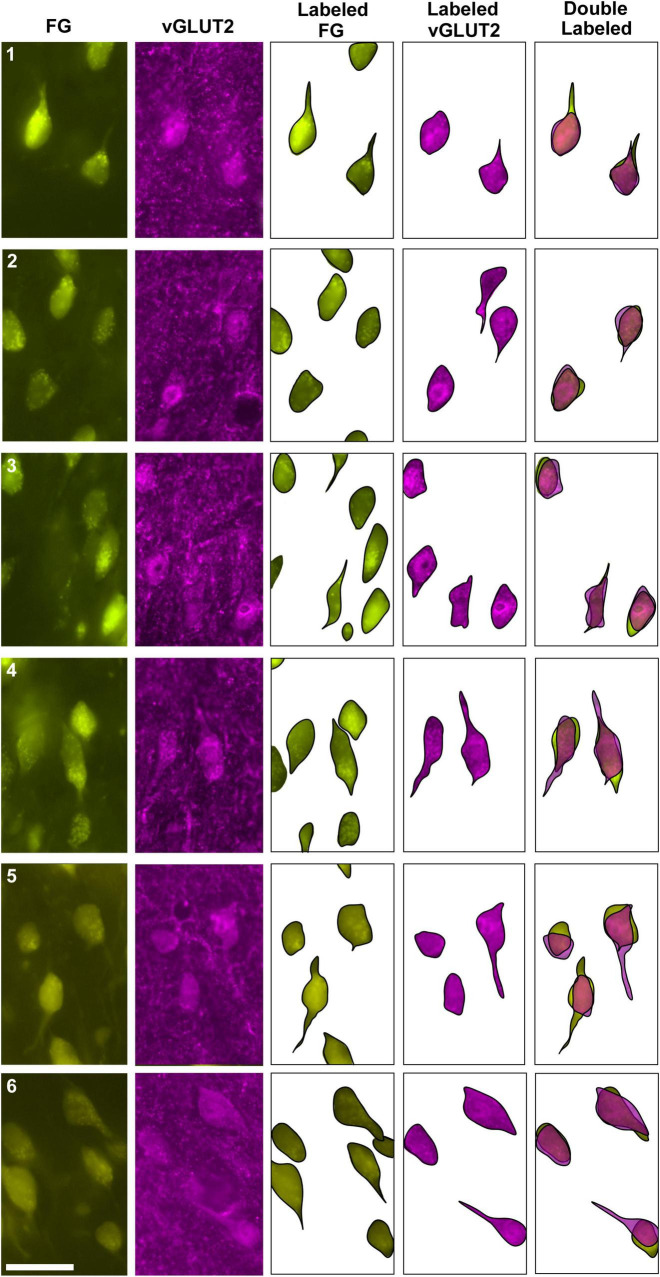
Photomicrographs (100x oil objective) and drawings of double-labeled LSO neurons (rows 1–6). Principal neurons were first retrogradely labeled via a unilateral injection of FG into the IC (column 1). This tissue was then stained by vGLUT2 antibodies (column 2) revealing that some FG neurons were co-labeled by a vGLUT2 antibody in either the ipsilateral or contralateral LSO. Not all principal neurons were double labeled. The neuronal shape of FG and vGLUT2 neurons from either the ipsilateral or contralateral LSO are shown (columns 3–4). Double-labeled neurons containing FG and vGLUT2 are identified by their overlapping position and near identical somatic features (column 5). Not all vGLUT2-stained neurons project to the IC, and not all IC-projecting neurons co-label with vGLUT2. Scale bar equals 25 μm.

Two different fluorescent filters were used for separately illuminating the IC-projecting neurons and the vGLUT2 neurons so that the images could be superimposed to reveal three types of neurons: (1) retrogradely labeled principal cells that co-labeled with vGLUT2; (2) principal cells that were vGLUT2 negative; and (3) vGLUT2 neurons that did not co-label with principal neurons projecting to the IC (Figures 5, 6). These neurons were observed bilaterally in the LSO.

### 3.3 Distribution of labeled LSO neurons

**FIGURE 7:**
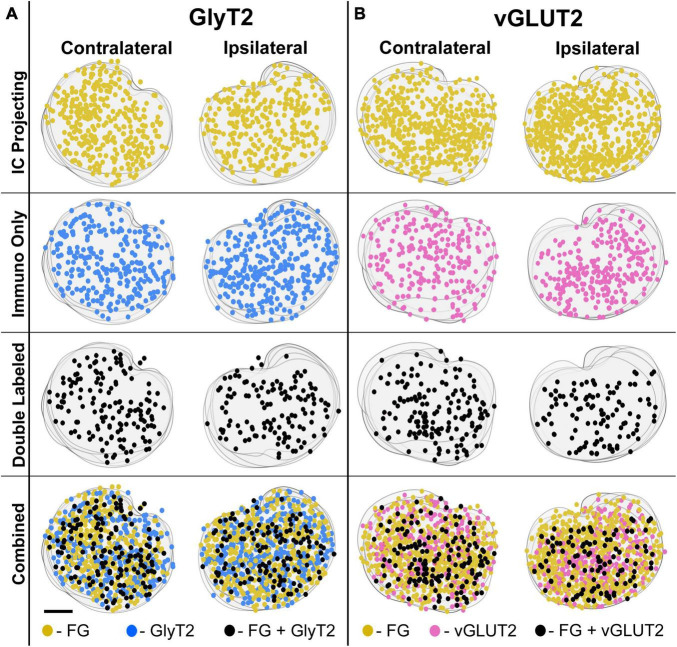
Representative IC-injection cases counterstained by GlyT2 antibodies or vGLUT2 antibodies demonstrate their bilateral distribution in the LSO. Neurons that project to the IC and/or are immunostained by GlyT2 **(A)** or vGLUT2 **(B)** antibodies were mapped onto outlines of their corresponding sections and collapsed in a z-stack for the contralateral and ipsilateral LSO. The distribution of labeled cells was relatively uniform and bilaterally symmetrical across the LSO for all cases, consistent with the tonotopic results of Williams et al. (2022). FG, FluoroGold; GlyT2, glycine transporter 2; LSO, lateral superior olive; CN VIII, vestibulocochlear nerve; IC, inferior colliculus; MNTB, medial nucleus of the trapezoid body; SOC, superior olivary complex. Scale bar equals 100 μm.

The LSO of representative IC injection cases, counterstained by GlyT2 and vGLUT2 antibodies, were drawn, aligned in register, and stacked in the z-plane. The positions retrogradely labeled LSO principal neurons, immunostained GlyT2 neurons, immunostained vGLUT2 neurons, and double-labeled neurons (FG and GlyT2 or FG and vGLUT2) were mapped. The results demonstrate that these various principal neurons are intermixed and distributed relatively uniformly throughout the LSO (Figure 7). The double-labeled neurons represent a smaller population compared to that of the IC projecting neurons or the GlyT2 and vGLUT2 immunolabeled neurons alone. Importantly, double-labeled IC projecting cells were observed in both the ipsilateral and contralateral LSO.

### 3.4 Counts of double-labeled neurons

The double-labeled neurons (FG and GlyT2 or FG and vGLUT2) were quantified by performing neuronal counts across serial sections from separate GlyT2 (*n* = 7) and vGLUT2 (*n* = 5) cases (Table 1). These cases received a single unilateral injection of a retrograde tracer, FG, into the IC. We observed an average of 704 ± 201.6 labeled neurons in the ipsilateral LSO and 701.6 ± 152.2 labeled neurons in the contralateral LSO. The spatial distribution of the projecting neurons in the LSO nuclei appeared symmetrical (Figure 7). This qualitative assessment was confirmed by the near-equal numbers of ipsilateral and contralateral projecting neurons whose ratio averaged near unity (Tables 1, 2A-projecting cells).

**TABLE 1:** Counts for LSO neurons in the ipsilateral and contralateral LSO nuclei.

Animal ID	Immuno label stain	Total IC projecting	IC projecting without immuno	Immuno label	Double labeled	% Double labeled
**Ipsilateral**						
AM1634	GlyT2	559	439	327	120	21.5
AM1643	GlyT2	603	415	485	188	31.2
AM1644	GlyT2	625	410	447	215	34.4
AM1655	GlyT2	651	494	468	157	24.1
AM1656	GlyT2	465	280	323	185	39.8
AM1657	GlyT2	661	487	381	174	26.3
AM1671	GlyT2	464	323	424	141	30.4
**Average ± SD**		575.4 ± 82.8	406.9 ± 79.8	407.9 ± 65.6	168.6 ± 31.8	29.7 ± 6.3%
**Contralateral**						
AM1634	GlyT2	619	542	352	77	12.4
AM1643	GlyT2	676	530	491	146	21.6
AM1644	GlyT2	558	401	427	157	28.1
AM1655	GlyT2	783	601	431	182	23.2
AM1656	GlyT2	582	356	415	226	38.8
AM1657	GlyT2	704	507	432	197	27.9
AM1671	GlyT2	446	304	369	142	31.8
**Average ± SD**		624 ± 109.7	463 ± 109.8	416.7 ± 45.7	161.0 ± 47.6	26.3 ± 8.3%
**Ipsilateral**						
AM1664	vGLUT2	1129	921	555	208	18.4
AM1665	vGLUT2	740	556	574	184	24.9
AM1671	vGLUT2	691	548	339	143	20.7
AM1672	vGLUT2	876	687	546	189	21.6
AM1673	vGLUT2	984	773	603	211	21.4
**Average ± SD**		884 ± 108.9	697 ± 156.6	523.4 ± 105.4	187.0 ± 27.2	21.4 ± 2.3%
**Contralateral**						
AM1664	vGLUT2	1020	791	593	229	22.5
AM1665	vGLUT2	703	509	576	194	27.6
AM1671	vGLUT2	699	541	319	158	22.6
AM1672	vGLUT2	732	528	591	204	27.9
AM1673	vGLUT2	897	694	588	203	22.6
**Average ± SD**		810.2 ± 42.8	612.6 ± 124	533.4 ± 120.0	197.6 ± 25.7	24.6 ± 2.8

Manual counts for LSO neurons labeled via IC retrograde injections and immune-histochemistry with GlyT2 (*n* = 7) or vGLUT2 (*n* = 5) were counted to reveal populations of single and double labeled neurons.

**TABLE 2:** Ratio between neurons in the ipsilateral and contralateral LSO nuclei labeled with either FG, GlyT2, vGLUT2, or double labeled.

Counts	Label	Ipsilateral	Contralateral	Ratio
**A Projecting cells**				
**Case**				
AM1634	FG	559	619	0.91
AM1643	FG	603	676	0.89
AM1644	FG	625	558	1.12
AM1655	FG	651	783	0.83
AM1656	FG	465	582	0.79
AM1657	FG	661	704	0.94
AM1671	FG	464	446	1.04
AM1664	FG	1129	1020	1.10
AM1665	FG	740	703	1.05
AM1671	FG	691	699	0.98
AM1672	FG	876	732	1.19
AM1673	FG	984	897	1.09
*Total*	−	8448	8419	1.00
**B GlyT2 cells**				
**Case**				
AM1634	GlyT2	337	352	0.96
AM1643	GlyT2	485	491	0.99
AM1644	GlyT2	447	427	1.05
AM1655	GlyT2	468	431	1.09
AM1656	GlyT2	323	415	0.78
AM1657	GlyT2	381	432	0.88
AM1671	GlyT2	424	369	1.14
*Total*	−	2865	2917	0.98
**C vGLUT2 cells**				
**Case**				
AM1664	vGLUT2	555	593	0.94
AM1665	vGLUT2	574	576	0.99
AM1671	vGLUT2	339	319	1.06
AM1672	vGLUT2	546	591	0.92
AM1673	vGLUT2	603	588	1.02
*Total*	−	2617	2667	0.98
**D Double labeled cells**				
**Case**				
AM1634	FG + GlyT2	120	77	1.56
AM1643	FG + GlyT2	188	146	1.29
AM1644	FG + GlyT2	215	157	1.37
AM1655	FG + GlyT2	157	182	0.86
AM1656	FG+ GlyT2	185	226	0.82
AM1657	FG + GlyT2	174	197	0.88
AM1671	FG + GlyT2	141	142	0.99
*Total FG + GlyT2*	−	1180	1127	1.05
AM1664	FG + vGLUT2	208	229	0.91
AM1665	FG + vGLUT2	184	194	0.95
AM1671	FG + vGLUT2	143	158	0.91
AM1672	FG + vGLUT2	189	204	0.93
AM1673	FG + vGLUT2	211	203	1.04
*Total FG + vGLUT2*	−	935	988	0.95

The ratio of labeled neurons between ipsilateral and contralateral neuronal counts was calculated for each case by dividing the ipsilateral count by the contralateral count. A ratio closest to 1.0 inferred symmetrical labeling between neuronal counts of both nuclei. **(A)** Principal neurons labeled via retrograde tracing in ipsilateral and contralateral LSO nuclei were counted. The total average ratio for the 11 cases is 1.0. Cases with alternate sections labeled with either GlyT2 or vGLUT2 and were counted for the principal neurons independently. **(B)** GlyT2 neurons labeled in the ipsilateral and contralateral LSO nuclei were counted. The average ratio of GlyT2 neurons between both nuclei was 0.98. **(C)** vGLUT2 neurons labeled in the ipsilateral and contralateral LSO nuclei were counted and the resulted in an average ratio equal to 0.98. The ratio of GlyT2 neurons between both nuclei was 0.98. **(D)** Doubled neurons were those retrogradely labeled neurons counterstained with either GlyT2 or vGLUT2. The average ratio of principal neurons double labeled with GlyT2 equalled 1.05; and the average ratio of principal neurons double labeled with vGLUT2 equalled 0.95.

The experiments on retrograde labeling coupled to transmitter immunocytochemistry were conducted in two separate series, separated in time by several months. We first made IC injections for the GlyT2 counterstaining (*n* = 7 mice), and after the resulting histology was finished, initiated IC injections for the vGLUT2 counterstaining (*n* = 5 mice). In spite of following the same procedures for both sets of experiments, injections from the second set resulted in a larger number of retrogradely-labeled cells compared to those of the first. As a result, we performed an ipsilateral:contralateral ratio analysis within each individual mouse rather than on raw counts; this strategy normalized the data from all the animals (Table 2). The IC-projecting GlyT2 neurons yielded an average ipsilateral:contralateral count ratio of 0.98 ± 0.29 (Table 2B), whereas the ipsilateral:contralateral count ratio for IC-projecting vGLUT2 neurons was 0.98 ± 0.08 (Table 2C). There was no statistical difference between these two sets of ratios (Mann Whitney two-tailed test, *p = 0.876*).

The IC-projecting neurons that doubled-labeled with GlyT2 exhibited bilateral symmetry in the labeling pattern across all seven cases, with a count ratio equal to 1.05 ± 0.23 (Table 2D). The average number of double-labeled neurons in the ipsilateral (GlyT2: 168.6 ± 31.8; vGLUT2: 187.0 ± 27.2) and contralateral (GlyT2: 161.0 ± 47.6; vGLUT2: 197.6 ± 25.7) LSO was also relatively consistent across animals (*n* = 12; Table 1) and revealed no statistical difference (Table 3, 2-way ANOVA).

**TABLE 3:** 2-WAY ANOVA results comparing projecting neurons double labeled with GlyT2 or vGLUT2 immuno-histochemistry.

Double label projecting cell comparison				
**Average % of double labeled cells**		**Mean Diff.**	***P*-value**	**Outcome**
GlyT2-ipsi	GlyT2-contra	3.4	0.88	ns
29.7	26.3			
GlyT2-ipsi	vGLUT2-ipsi	7.8	0.24	ns
29.7	21.4			
GlyT2-ipsi	vGLUT2-contra	3.2	0.94	ns
29.7	24.6			
GlyT2-contra	vGLUT2-ipsi	4.4	0.79	ns
26.3	21.4			
GlyT2-contra	vGLUT2-contra	−0.17	>0.99	ns
26.3	24.6			
vGLUT2-ipsi	vGLUT2-contra	−4.6	0.79	ns
21.4	24.6			

Comparisons were made between the ipsilateral and contralateral LSO nuclei and between stains. The results revealed no significant difference across all comparisons. Data expanded from Table 1. ipsilateral, ipsi; contralateral; contra.

**FIGURE 8:**
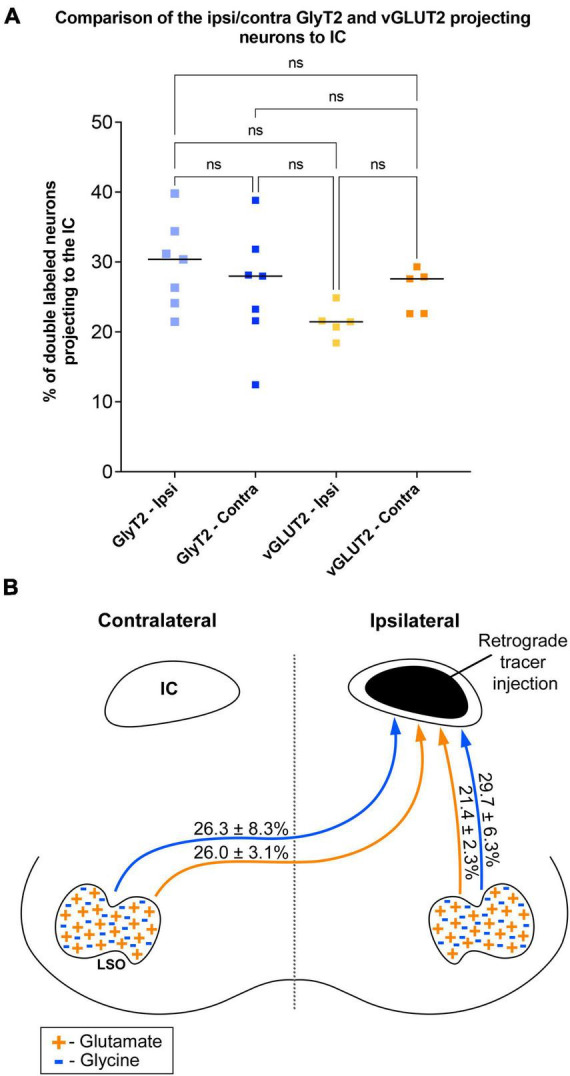
Summary of normalized numbers of GlyT2- and vGLUT2-projections to the IC. **(A)** Counts of double-stained neurons were normalized for each animal against the total number of IC projecting cells due to uncontrollable individual differences as well as variations in IC injections placement and size, retrograde transport efficiency, sensitivity of antibody lots, and degree of tissue fixation. The plot confirms what histological analyses suggested: there is no statistical difference between the relative numbers of GlyT2 and vGLUT2 staining of LSO principal cells or between their ipsilateral and contralateral projections to the IC. **(B)** Graphic illustration of the projection ratios of GlyT2 and vGLUT2 to a single IC. The inputs to the opposite IC are not shown but would be a mirror image.

The symmetry in the labeling pattern for GlyT2 double-labeled projecting neurons was further evident across all cases by dividing the number of double-labeled neurons in the ipsilateral nucleus by the total number of IC projecting neurons in the ipsilateral nucleus, which revealed 29.7 ± 6.3% of neurons in the ipsilateral LSO were co-labeled and 26.3 ± 8.3% of neurons in the contralateral LSO were co-labeled (Table 1). The double-labeled neurons were intermixed with single-labeled projecting neurons and single-labeled GlyT2 neurons. There was no significant difference in the percentages when comparing the co-labeled neurons in the ipsilateral versus the contralateral LSO (2-way ANOVA, *p = 0.88*; Figure 8A and Table 3).

When double-labeled vGLUT2 neurons were averaged across five cases, the ipsilateral LSO contained 21.4 ± 2.3% IC-projecting neurons compared to the 24.6 ± 2.8% contralateral IC-projecting neurons (Table 1). No significant difference was found in the percentages of double-labeled vGLUT2 neurons with ipsilateral or contralateral projections (2-way ANOVA, *p* = 0.79; Figure 8A and Table 3). In a similar way, the percentages of doubled-labeled GlyT2 projecting neurons and vGLUT2 projecting neurons in the ipsilateral and contralateral LSO nuclei also revealed no significant differences (Table 3).

### 3.5 Cresyl violet staining features

In CV-stained material, the cytoplasm of principal cells lacked large stacks of rough endoplasmic reticulum, also known as Nissl bodies. Free ribosomes, however, were plentiful and gave the cytoplasm a fine, granular light-blue texture. A pale spherical nucleus with a single nucleolus occupied the middle of the spindle-shaped cell body. Views of the principal cell away from its center-of-gravity often missed the pointed ends of the spindle but revealed an oval cell body dominated by the presence of the central nucleus (Williams et al., 2022).

Counts in CV material were made according to four types of cells identifiable in the tissue (Supplementary Figure 3 and Supplementary Table 1): principal cell with nucleus present, principal cell with no nucleus present, small cell with nucleus present, small cell with no nucleus present. A total of 2277 neurons were counted in the left LSO, and a total of 2785 counted in the right LSO. For the IC projecting neurons, we counted 703 ± 174.7 labeled neurons per LSO. From previous work (Williams et al., 2022), there is an average of 362 ± 25.4 lateral olivocochlear efferents and from this study, an average of 412 ± 54.5 GlyT2 and 528 ± 106.6 vGLUT2 neurons in each LSO nucleus. The sum of the average number of IC-projecting neurons, LOCs, GlyT2 and vGLUT2 neurons in one LSO equals 2005. The projection pattern of these neurons is summarized in Figure 8B.

### 3.6 Soma silhouette area of the labeled neurons

**FIGURE 9:**
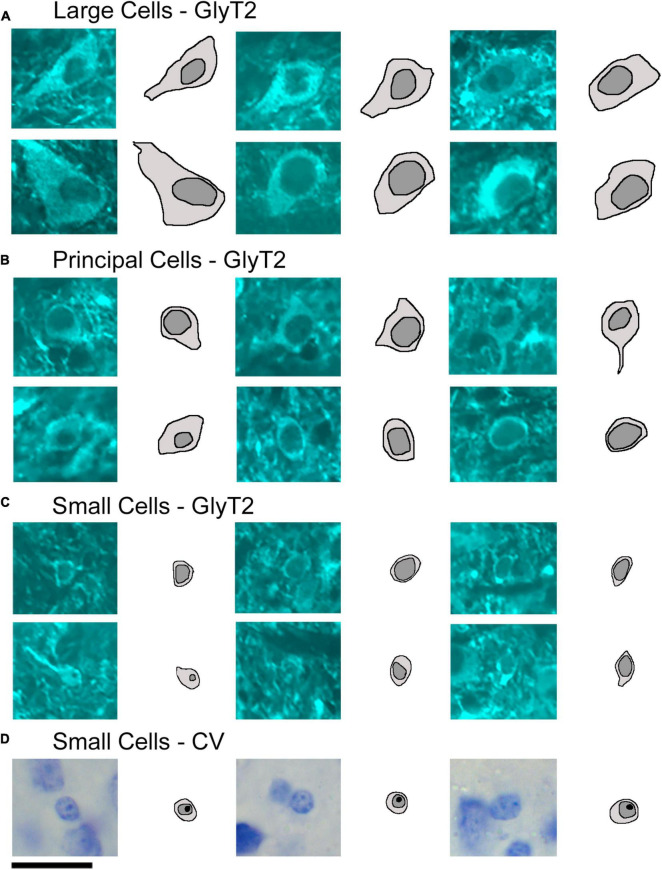
Photomicrographs (100x oil) with corresponding drawings of large, medium, and small GlyT2-labeled neurons of the LSO. GlyT2 immunostaining revealed a small population of large and small GlyT2-labeled cells that were intermixed with the dominant, medium-sized principal neurons. GlyT2 neurons were traced to illustrate the cell body silhouette and its resident nucleus. **(A)** Large GlyT2-labeled neurons were sprinkled around the borders of the nucleus and the dorsal hilus. These border neurons were similar in size, shape, and location to previously described periolivary neurons (Williams et al., 2022). **(B)** Medium-sized GlyT2-labeled neurons met the criteria of principal neurons. **(C)** The cell bodies of the small GlyT2-labeled neurons were round-to-oval in shape and never labeled by retrograde tracer injections in the IC. **(D)** CV staining revealed small cells to have granular and slightly lumpy cytoplasm and a round nucleus with pale grainy chromatin and prominent nucleolus. Scale bar equals 25 μm.

Neuronal size variations were observed from the different sets of stained tissue−LSO projecting neurons revealed qualitative medium and large neurons; GlyT2-labeled neurons were small, medium, and large (Figures 9A–C); and vGLUT2-stained neurons were medium and large. These qualitative observations were confirmed in quantitative analyses using soma silhouette area. The data confirmed a small population of small glycinergic neurons that were revealed by GlyT2 antibodies and related to small LSO neurons stained by CV (Figure 9).

Analysis of soma silhouette area was used to analyze neuron size groups in the LSO (Supplementary Figure 6). In our previous study (Williams et al., 2022), we determined that the principal neurons have an average cell area of 123.9 ± 26.6 μm^2^, comparable to medium sized GlyT2- and vGLUT2-labeled neurons in this study.

GlyT2 neurons could be classified into three size categories (Figure 9): large cells, which had polygonal somata and resided around the borders of the LSO; medium-sized neurons, corresponding to descriptions of the principal cells with fusiform somata and unipolar or bipolar dendritic extensions; and small cells featuring somata that were < 40 μm^2^, oval, and containing a pale spherical nucleus.

**FIGURE 10:**
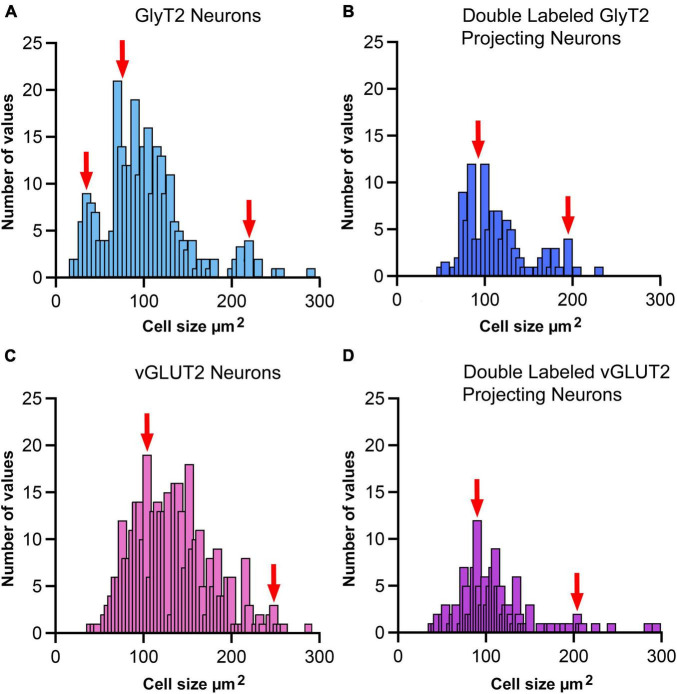
Cell size histogram for labeled LSO neurons. The outlines of cell bodies exhibiting a distinct nucleus were drawn from photomicrographs (100x oil) so that somatic silhouette area and shape could be calculated: **(A)** GlyT2 immunostaining, **(B)** double-labeled by FG and GlyT2, **(C)** vGLUT2 immunostaining, and **(D)** double-labeled by FG and vGLUT2. (A) GlyT2 labeling revealed three population based on somatic size: small, medium and large, which aligned with qualitative descriptions provided in Figure 9. A histogram of somatic area showed three peaks (red arrows) that were consistent with three populations based on somatic size. Panel **(B)** Somatic size differences revealed two population peaks (red arrows) for GlyT2 neurons that projected to the IC: medium-sized and large neurons. No small GlyT2 neurons were observed projecting to the IC. Panel **(C)** vGLUT2 antibodies labeled medium-sized and large neurons in the LSO; these two populations resembled the previously described LSO principal and PO neurons, respectively. No small neurons were observed in the LSO stained by vGLUT2 antibodies. Panel **(D)** Neurons double-labeled by FG and vGLUT2 antibodies revealed two population peaks (red arrows) for medium-sized and large neurons attributed to the principal and periolivary neuronal classes. These histograms demonstrate that principal/medium-sized neurons dominate the LSO but that small and large neurons are also present. Histogram bin width equalled 5 μm2.

Soma silhouette values for GlyT2 neurons were consistent with a tri-modal distribution (Figure 10 and Table 4): small GlyT2 neurons had an average cell size equal to 37.73 ± 8.30 μm^2^, the medium sized neurons had an average size equal to 100.9 ± 25.54 μm^2^, and the large neurons had an average cell size equal to 215.0 ± 48.46 μm^2^ (Supplementary Figure 6A). In the population of LSO-projecting neurons that co-labeled with GlyT2, these small neurons of the mouse LSO had not been previously described.

**TABLE 4:** Representative cell size of GlyT2, vGLUT2, and projecting neurons, and double labeled neurons.

GLYT2							
	**GlyT2 (small)**	**GlyT2 (medium)**		**GlyT2 (large)**		**GlyT2 IC projecting (medium)**	**GlyT2 IC projecting (large)**
Number of cells	38	212		18		94	18
Area median (μ m^2^)	38.2	99.5		208.7		100.5	180.8
Area mean ± standard deviation (μ m^2^)	37.73 ± 8.3	100.9 ± 25.5		215.0 ± 48.5		99.14 ± 22.9	180.8 ± 21.2
**vGLUT2**							
	**vGLUT2 (medium)**		**vGLUT2 (large)**		**vGLUT2 IC projecting (medium)**		**vGLUT2 IC Projecting (large)**
Number of cells	346		38		122		16
Area median (μ m^2^)	123.3		222.8		99.4		200.7
Area mean ± SD (μ m^2^)	114.0 ± 40.6		230.4 ± 42.2		103.1 ± 24.8		202.7 ± 44.0
**FG**							
	**IC projecting principal cells (medium)**				**IC projecting principal cells (large)**		
Number of cells	551				64		
Area median (μ m^2^)	95.9				148.0		
Area mean ± SD (μ m^2^)	96.9 ± 22.5				157.2 ± 28.4		

Labeled LSO neurons were divided into categories based on their labeling technique and drawn and measured for cell size silhouette area (μm^2^) from multiple cases. GlyT2 featured a population of small neurons that were not seen in vGLUT2 material.

Neurons labeled with vGLUT2 antibodies revealed a relatively uniform group of cells that resembled the descriptions of the principal neurons. These neurons were medium sized, averaging 114.0 ± 40.61 μm^2^ (Figure 10). A small population of large polygonal neurons were observed around the LSO borders and fit the descriptions of PO neurons (mean 230.4 ± 42.15 μm^2^; Table 4 and Supplementary Figure 6B). The IC-projecting neurons that co-labeled with vGLUT2 showed similar sizes to vGLUT2 neurons that did not project to the IC. No small neurons were stained by vGLUT2 antibodies.

### 3.7 Summary

The present findings demonstrate that the population of principal cells of the LSO include bilateral and symmetric projections of glycine and glutamate cells to the IC in the CBA/CaH mouse. We previously documented that these projections are tonotopic (Williams et al., 2022). The organization of these projections to the IC add to our knowledge of how excitation and inhibition contribute to the separate binaural processing demands for localizing high and low frequency sounds. We also observed that not all GlyT2- or vGLUT2-labeled neurons project to the IC: these must project to other brain stem sites, such as the cochlear nucleus, superior olivary complex, nuclei of the lateral lemniscus, or thalamus.

## 4 Discussion

The ability to determine the spatial location of a sound is a remarkable accomplishment of the ears and brain. The localization of a sound source is computed by acoustic cues that are created by the physical interactions of the sound with the head and the two ears, including pinna and ear canals. The cues are analyzed by the central auditory system, which uses the neural signals to create a representation of auditory space. The practical consequence of this ability is to avoid unseen dangers, to orient to the sounds of a potential mate or foe, and to separate simultaneously occurring acoustic streams.

Two ears are clearly important because differences in timing and intensity of arrival of sound at the ears provide binaural cues for sound localization in the azimuthal plane (Jeffress, 1948; Boudreau and Tsuchitani, 1970; Guinan et al., 1972; Grothe, 2000; Konishi, 2000). In the classic model, low frequency sounds produce binaural timing differences, whereas high frequency sounds yield interaural sound level differences (Stevens and Newman, 1936). In addition, the reflections of sounds off the head and pinna and within the ear canals create spectral cues crucial for distinguishing sound distance, elevation, and front-back positioning (Reiss and Young, 2005).

Interaural distance, the distance between the two ears, is a factor that is related to LSO development and to the range of sound localizing abilities across species (Masterton et al., 1969). In a general way, animals with smaller heads have better sound localizing abilities but other factors such as animal niche and considerations of predator vs. prey also play a role. A relationship between interaural distance and high frequency hearing has been noted, but there are exceptions (Irving and Harrison, 1967; Masterton et al., 1969; Moore and Moore, 1971; Heffner and Masterton, 1990). The LSO is a major nucleus in the SOC that is involved in transmitting binaural auditory signals to higher structures and controlling cochlear receptor sensitivity via its descending projections (Dewson, 1967; Liberman, 1980; Darrow et al., 2006; Malmierca and Ryugo, 2011). All extant animals are specialized and adapted to survive in their particular environmental niche, whether it be by reproduction strategies, predation, or complex social systems (Dunbar, 2009). These specializations will be reflected in brain anatomy and physiology.

### 4.1 Cell types and neurotransmitters

LSO principal neurons that project to the IC are crucial for modeling the role of excitation and inhibition in binaural hearing (Finlayson and Caspary, 1991; Glendenning et al., 1992; Henkel and Brunso-Bechtold, 1993; Brunso-Bechtold et al., 1994; Franken et al., 2018). Not surprisingly, the exploration of cell types and neurotransmitter expression involved in the auditory pathways have been a subject of extensive study over the years and across species including cats, mice, rats, humans, and ferrets (Adams, 1979; Ollo and Schwartz, 1979; Glendenning and Mastereton, 1983; Cant, 1984; Helfert and Schwartz, 1986, 1987; Helfert et al., 1989; Eybalin, 1993; Henkel and Brunso-Bechtold, 1993; Rietzel and Friauf, 1998; Kulesza, 2008).

Generalities regarding cell types have been impeded by variations in cell staining such as *basophilic and silver proteinate dyes* (Taber, 1961; Irving and Harrison, 1967; Fech et al., 2017), *histochemistry* (Warr, 1975), *immunocytochemistry* (Storm-Mathisen et al., 1983; Wenthold et al., 1986; Vetter et al., 1991; Helfert et al., 1992; Berrebi and Spirou, 1998; Kulesza, 2014; Williams et al., 2022), and *Golgi techniques* (Scheibel and Scheibel, 1974; Ollo and Schwartz, 1979; Rietzel and Friauf, 1998). The Golgi-method has had more limited utility because of its preference to work in younger animals (Ryugo and Fekete, 1982). Different staining methods inherently require separate criteria for names because different stains are designed to reveal distinct features of the neurons. Cross validation for different methods has only become available recently with the advent of immunocytochemical double-labeling procedures.

Studies also used different *pathway tracing* techniques that varied in sensitivity and therefore in reliability. *HRP histochemistry* (Adams, 1979; Schweizer, 1981; Nordeen et al., 1983; Glendenning et al., 1992) was the first true neuronal tracer (LaVail et al., 1973) and its sensitivity was dependent on a variety of conditions, particularly on the chromogen used (Mesulam, 1978; Adams, 1981). The discovery that biotinylated dextran amines could be transported along axons and finely visualized by reactions with diaminobenzidine represented a crucial advance in pathway tracing sensitivity (Reiner et al., 2000). This new method became the standard for identifying pathways in the auditory brain stem (Loftus et al., 2004; Gómez-Álvarez and Saldaña, 2016; Williams et al., 2022), but could be replaced by the constantly evolving technology that has introduced a perhaps even more selective and sensitive method of axon tracing using viral vectors (Lanciego and Wouterlood, 2020; Qiu et al., 2022). A less common method using the *selective uptake and transport of radiolabeled glycine* (Saint Marie and Baker, 1990; Glendenning et al., 1992) and *amino acid receptor immunolabeling* (Koch and Sanes, 1998) have contributed to the literature on cell types, potential transmitter, and cell type location in the LSO. All of these various methods fundamentally require replication using different methods but in the same species. In short, global conclusions regarding cell types, transmitter chemistry, and IC projections have been generally hampered by studies using different species, ages, methods, and naming criteria.

**FIGURE 11:**
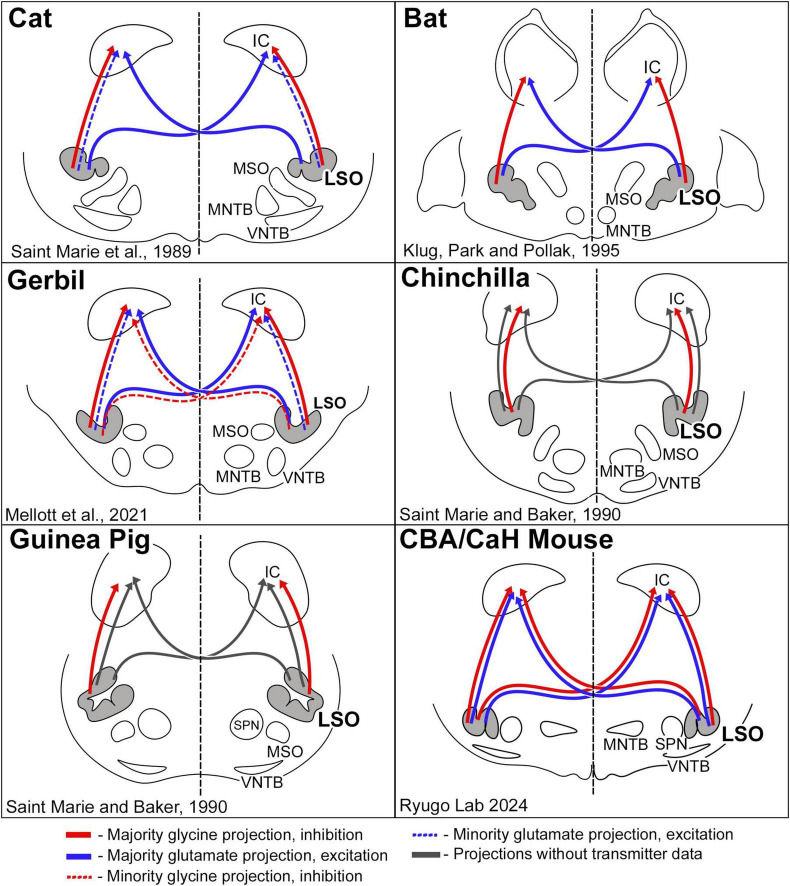
Schematic diagram of excitatory and inhibitory projections from LSO cells as reported for a few species of mammals. The pattern of glycinergic and glutamatergic projections in the cat suggested that inhibitory projections were entirely ipsilateral, and that excitatory projections were predominately contralateral (Saint Marie et al., 1989). This pattern was essentially replicated in the bat (Klug et al., 1995). The gerbil resembled the cat, with the additional observation that there was a small group of glycinergic neurons that projected to the contralateral IC (Mellott et al., 2021). The chinchilla and guinea pig featured only ipsilateral glycinergic projections; no glutamate labeling was shown. Contralateral projections were shown by retrograde transport of horseradish peroxidase but without transmitter information (Saint Marie and Baker, 1990). In the CBA/CaH mouse, we observed a bilateral and symmetric projection to the IC for both glycinergic and glutamatergic LSO neurons. These variations in projection patterns are hypothesized to reflect processing differences forged by adaptive mechanisms for different habitats and survival demands.

The investigation of neurotransmitter expression, particularly regarding glycine and glutamate, has yielded general agreement where the ipsilateral projection from the LSO to the IC was inhibitory and glycinergic and the contralateral projection from the LSO to the IC was excitatory and glutamatergic (Figure 11). There were, however, differences regarding the extent of contralaterally projecting glycinergic neurons and/or ipsilaterally projecting glutamatergic neurons (Helfert et al., 1989; Saint Marie et al., 1989; Saint Marie and Baker, 1990; Klug et al., 1995; Cant and Benson, 2006; Mellott et al., 2021; Haragopal et al., 2023). In studies that did not analyze cell chemistry, projections were reported as bilateral and symmetrical (Adams, 1979; Schweizer, 1981; Nordeen et al., 1983; Ross et al., 1988; Grothe, 1994; Kelly et al., 1998). We have shown in the CBA/CaH mouse that glycinergic and glutamatergic principal cells of the LSO project bilaterally, symmetrically, and topographically to the IC (Williams et al., 2022; present report).

A comment of cell chemistry seems in order. It has been reported that vGLUT2 is found only in a subset of “nerve terminals” whose distribution appears sorted by “level” along the neuraxis. However, the expression and distribution might also be dependent on the excitatory state or the neuron’s packaging/release properties at the time (Fremeau et al., 2004). It has also been reported that vGLUT2 is present in cell bodies (Fremeau et al., 2001; Li et al., 2020). Since vGLUT2 is reported to be distributed extrasynaptically (Gomeza et al., 2003), it is not surprising that it has also been observed in LSO principal cells using immunostaining (Blaesse et al., 2005; Ito and Oliver, 2010) or *in situ* hybridization (Ito et al., 2010). There remains much to be learned about vGLUT2—its synthesis, regulation, trafficking, and activity-dependence (Martinez-Lozada and Ortega, 2023). In our hands, the vGLUT2 antibody behaves like a specific marker for glutamatergic LSO neurons, which was our goal in the first place.

There is also an issue as to the distribution of GlyT2 staining. GlyT2 has been reported to be primarily in terminal endings (Altieri et al., 2014; Gessele et al., 2016; Mellott et al., 2021), although in our work, GlyT2 antibodies clearly label what may be interpreted as glycinergic cell bodies. Neurons of the MNTB are known to be glycinergic (e.g., Helfert et al., 1989), and MNTB somata stain prominently in our material using GlyT2 antibodies (Supplementary Figure 2; Milinkeviciute et al., 2017 using a CBGlyT2-EGFP mouse). The MNTB staining serves as a positive control for our LSO staining. This staining of MNTB and LSO neurons using GlyT2 antibodies is also consistent with images shown by others (Friauf et al., 1999; Ngodup et al., 2020). These variations may be related to why the contralateral glycinergic projections from the LSO to the IC was not observed in the C57BL/6 mouse (Haragopal et al., 2023) or the gerbil (Mellott et al., 2021). Alternatively, the variations may be strain-specific or due to differences in technique.

### 4.2 Excitation and inhibition

The physiological features of LSO cells have been featured by no activity in the absence of sound, excitatory responses from ipsilateral sounds, and suppressed activity by contralateral sounds. Single-unit, extracellular recordings revealed the narrow, V-shaped excitatory and inhibitory tuning curves with similar characteristic frequencies (Tsuchitani and Boudreau, 1966, 1967; Guinan et al., 1972). While the LSO is best known for processing interaural level differences, the inhibitory effects of the contralateral ear are most pronounced when the stimuli are closely matched in frequency (Boudreau and Tsuchitani, 1968, 1970; Tollin and Yin, 2002) although there are more recent data suggesting that LSO neurons are also sensitive to the timing of sound onset (Franken et al., 2018), sound envelope emphasizing interaural time differences (Joris and Yin, 1998), or timing information extracted from binaural interactions (Benichoux et al., 2018). These data clearly reveal that the binaural balancing of excitation and inhibition for incoming signals in the LSO is influenced by the various spectral and temporal properties of the sound.

Excitation of a cell by presenting a CF tone in the ipsilateral ear increases as the intensity with which that sound is played increases until reaching a plateau. In parallel, contralateral inhibition measured by a decrease in spikes occurs with increasing intensity of the sound. It is the intensity difference between the ipsilateral and contralateral sound that will elicit or inhibit the spike output. The LSO is equipped to carry out differentiation of incoming sounds based on their level differences while the frequency involvement of this phenomenon remains vital (Goupell and Stakhovskaya, 2018). The convergence of excitatory and inhibitory inputs within the LSO will determine how principal cells acquire their sensitivity for binaural level and time differences.

### 4.3 Projections

The establishment of excitatory and inhibitory neurons in the LSO represents the next stage in the mechanics of sound localization. It is at this level where excitatory inputs converge onto certain types of neurons in the LSO, leading to the initiation of second-order excitatory and inhibitory projections. The distribution of these projections is important for thinking about how auditory space is constructed, at least in terms of following how ipsilateral ear excitation becomes transformed into binaural responses that convey excitation or inhibition.

When considering the nature of IC-projecting principal cells, approximately 25% of them were double labeled bilaterally by GlyT2 or vGlUT2. We did not perform double immunolabeling with the projection experiments, but if we consider both types of staining separately, half of the IC-projecting cells were labeled by GlyT2 or vGLUT2. This situation implies that the other half of the IC-projecting cells are not using glycine or glutamate. Another consideration must deal with the GlyT2 and vGLUT2 immunolabelled cells that do not appear to project to the IC. We need to know what the chemistry is for these cells and where they project.

In the mouse, it would appear that the mechanisms for binaural processing between the LSO and IC could have a basic repetitive organization. Such a prediction is based on our observation that projections of principal cells are bilateral, topographic, and symmetrical. As such, we propose that a binaural spatial processing unit in the IC could be modeled by bilaterally-matched pairs of isofrequency laminae. This idea is consistent with observed on-going binaural disparity in frequency-intensity spectra as one of the cues for determining the direction of a sound, especially in species with a small head (Harper and McAlpine, 2004; Harper et al., 2014) or a small or absent medial superior olive (Irving and Harrison, 1967; Masterton et al., 1967). The test of such a hypothesis still requires new knowledge about brain stem circuits of the auditory system (Glendenning and Masterton, 1998) and how the various ascending projections are synaptically arranged with the multiple cell types comprising an IC lamina (Oliver, 2000; Loftus et al., 2004, 2010).

An important issue is to understand the differences in results reported for the C57BL/6 mouse (Haragopal et al., 2023) versus what we report for the CBA/CaH mouse. One possibility concerns the mouse strain: the use of the C57BL/6 strain is advantageous for genetic manipulations (Hasan et al., 2004; Hawrylycz et al., 2011) but perhaps not for hearing research. C57BL/6 mice progressively lose hearing starting at 2 months of age (Henry and Chole, 1980; Willott et al., 1985; Ison and Allen, 2003), and high frequency hearing loss starts as early as 6 weeks of age (Ouagazzal et al., 2006). In addition, auditory efferents decline rapidly after 6 weeks of age (Zhu et al., 2007, and there is evidence for central compensatory plasticity Willott and Turner, 1999). In contrast, the stable hearing thresholds of CBA/CaJ mice over time provide a more reliable model and reference for normal hearing mouse strains (Zheng et al., 1999).

There are also technical details in the Haragopal et al. (2023) publication that should be considered. The IC injection volume of only 80 nl of Fluoro-Ruby may not be sufficient to yield a reliable labeling pattern vis-à-vis the contralateral and ipsilateral distribution. The injection site in their (Figure 2) does not include the lateral third of the CNIC. Our pressure injection volumes were significantly greater. In addition, the use of *in vitro* hybridization is reported to weaken the fluorescent signal of their retrograde tracer, Fluoro-Ruby, but the authors do not show the effectiveness of the anti-TRICT retrieval treatment or include *in situ* hybridization controls. The authors do not provide criteria for what they consider labeling nor provide photographic evidence for such labeling. The low magnification of their photomicrographs and undefined arrows and arrowheads likewise raise issues in their cell counts and the ipsilateral/contralateral nature of their projections.

There remains the question for how the “what” component of sound is integrated with the “where” component (Romanski et al., 1999; Cloutman, 2013; Rauschecker, 2018). Binaural networks are important in dynamic sound processing. Proprioceptive, visual, and vestibular systems provide additional information about head and body position, movement, and gravity, and as such, contribute significantly to the “where” component (Rice et al., 1992; Wright and Ryugo, 1996; Kanold and Young, 2001; Kanold et al., 2011). These multisensory systems establish and maintain on-going relationships between sound sources and the position of the listener in space (Ryugo et al., 2003; Wu and Shore, 2018; Ansorge et al., 2021; Ryugo and Milinkeviciute, 2023). Understanding how these sensory circuits and their cellular constituents work together are key to grasping how mammals cope in an acoustic environment where sound is constantly changing in spectral-temporal features, loudness, and position.

### 4.4 LSO counts

In our study, we performed unilateral IC injections, which labeled neurons in the LSO that double labeled with approximately 20–30% of neurons labeled with either GlyT2 or vGLUT2 in the ipsilateral or contralateral nuclei. We would expect that for bilateral IC injections, the number of double labeled neurons in each LSO for GlyT2 and vGLUT2 cases would increase by twofold. When trying to resolve the overall number of neurons in the LSO, we must consider not just the principal cells, or those that project to the IC, but also the principal neurons who project DNLL or CN and the LSO efferents that project to the ipsilateral cochlea.

The number of AChE-stained cells of the LSO was concluded to represent the total number of LOC neurons because the number of retrogradely labeled, HRP-neurons equaled the number of AChE-stained neurons in adjacent sections and because HRP labeled neurons showed close correspondence to AChE staining when double-labeling methods were used (Warr, 1975). Similar results were found in the CBA/CaH mouse using the retrograde tracer, FluoroGold, with AChE and ChAT (Suthakar, 2017; Williams et al., 2022). These results imply that cholinergic staining of LOC efferents avoids the question of incomplete marking of LOCs due to inadequate access to the tracer.

The relative presence of other neuroactive substances In LOC neurons such as GABA, glycine, dopamine, dynorphin, and nitric oxide seems to vary with age, species, method of staining, laterality of projection, and history of noise exposure (Helfert et al., 1989; Vetter et al., 1991; Vetter and Mugnaini, 1992; Kandler et al., 2002; Maison et al., 2002; Nabekura et al., 2003; Schaeffer et al., 2003; Niu et al., 2004; Jenkins and Simmons, 2006; Wu et al., 2020). In our hands, the retrograde labeling of LOC efferents using FG injections in the cochlea did not co-label with GAD67 positive cells from our transgenic GAD67/EGFP mice (Suthakar, 2017) or with GAD67 positive immunostained cells in CBA/CaH mice. A summary of LSO neuronal counts across studies and involving different labels is provided in Supplementary Table 1.

### 4.5 Consideration of methods of study

The variety of results that accompany differences in species, age, habitat, and hearing range and sensitivity could provide important links to the study data and some variable that might not have been previously considered, perhaps yielding novel insight into its neural substrate. In our study, we attempted to label LSO neurons using a CBGlyT2-EGFP (Zeilhofer et al., 2005) backcrossed > 10 generations with CBA/CaH mice but could not detect label in LSO neurons. Our attempts to label LSO neurons in the mouse with antibodies against vGLUT1 was also unsuccessful, whereas this method did stain LSO neurons in the rat (Ito and Oliver, 2010). We were unable to determine if the CBA/CaH mouse LSO did not use vGLUT1 transporter or if the antibody or our method was flawed. Superimposed on the inherent differences between species is the fact that different research methods will also yield different kinds of data: (1) intact animal versus *in vitro* slice preparations for physiologically characterizing cell properties; (2) basic dyes, immunocytochemistry, *in situ* hybridization, or transgenic animals for specific cell staining; (3) age of subject for examining growth and development; (4) anterograde, retrograde or viral tracing for describing neuronal circuits; and (5) microscopic visualization technique (brightfield, fluorescent, confocal, light sheet, multiphoton, scanning and electron microscopy). Simple things like staining artifacts—fixation and tissue preservation, tissue distortion while staining, tissue stretching when mounting sections on microscope slides, tissue shrinkage when dehydrating sections for coverslipping−all contribute to microscopic changes. By recognizing the potential sources of variability inherent to any study, we will be better prepared to interpret comparative data.

### 4.6 Species differences

It has been shown that different species exhibit varying immunochemical and IC-projection characteristics related to the frequency response properties of the neurons under study (Saint Marie et al., 1989; Brunso-Bechtold et al., 1994; Barnes-Davies et al., 2004; Mellott et al., 2021). There are reports of a low frequency bias for ipsilateral projections to the IC and a high frequency bias for contralateral projections (*cat*, Glendenning and Mastereton, 1983; *gerbil*, Mellott et al., 2021). A quite different conclusion was reached in the ferret where the laterally-situated low frequency neurons preferentially projected to the contralateral IC, whereas the medially-situated high frequency neurons projected to the ipsilateral IC (Henkel and Brunso-Bechtold, 1993). Because cats and ferrets are both small carnivoran species and potent predators, such differences might not be expected. Species by itself, however, does not ensure trait uniformity: there are many different strains of mice that are specialized for one feature or another (e.g., Fontaine and Davis, 2016; Smith, 2019).^1^ The available data suggest that there is still much to be learned about brain size and circuits as they relate to evolution, species, habitat, and behavior.

Perhaps we need to consider additional details that influence body anatomy, behaviour, reproduction, and ecology. For example, cats and ferrets are both carnivorous mammals with flexible body structure adapted for hunting (Barratt, 1997; Marshall, 2020), but belong to different families: cats are felines, whereas ferrets are weasels. Cats are solitary hunters with distinct predatory behaviors (Marshall, 2020) and are found in various habitats, occupying a wide range of ecological niches (Miller, 1996), not unlike that of ferrets (McKay, 2012). It is of some interest that the cat was domesticated some 10,000 years ago (Driscoll et al., 2009). In contrast, the ferret was domesticated roughly 2,000 years ago (Davison et al., 1999), providing less time for environmental pressures to induce brain and behavior changes.

Mice and rats are small rodents, herbivorous, and thigmotaxic with whiskers that guide them to walls or other points of hiding (Harris, 1979; Traweger et al., 2006). They belong to the same taxonomic family, live in colonies, occupy simple burrows, and display complex social behaviors in fields, forests, or domestic regions (Rossi, 1975; Ehret and Riecke, 2001; Bonthuis et al., 2010; Hikishima et al., 2017; Netser et al., 2020). However, rats are considered natural predators of mice (Liu et al., 2017), and predator-prey relationships are suspected to contribute to differences in brain circuitry (Apfelbach et al., 2005). Gerbils live in family groups, are gregarious, and known for their more elaborate burrows and burrowing behavior (Fisher and Llewellyn, 1978). Because of these variations in habitat and behavior, we can infer that they have an impact on how each species processes and locates sound.

The natural habitats of the chipmunk, gerbil, and kangaroo rat are moderately elaborate underground burrows, whereas the rat and the mouse are known to create shallow and somewhat simple burrows (Supplementary Figure 7; Storer, 1948; Elliott, 1978, Avenant and Smith, 2003; Scheibler et al., 2006; Weber et al., 2013; Vorhies and Taylor, 2015). In marked contrast, the naked mole rat lives in complex burrows, highly branched with up to 6 km of total tunnel length and extending across as much as 6 football fields (Buffenstein et al., 2012; Park and Buffenstsein, 2012). In burrows, the transmission of high frequency sounds is significantly reduced and the need for sound localization is generally limited to front-back distinctions (Heth et al., 1986; Begall et al., 2007; Okanoya et al., 2018; Barker et al., 2021). It should not be surprising that the auditory system of burrowing animals differs from that of above-ground mammals, and these differences can be reflected their audiograms (Supplementary Figure 8) as well as how sound is processed by the LSO (Moore and Moore, 1971). The differences between common auditory research subjects such as gerbils, guinea pigs, chinchillas, cats, and mice could be associated with anatomical specializations that underlie mechanisms of binaural processing.

Differences in results and/or conclusions presented in published reports are worthy of additional mention. In terms of comparative neurobiology, there is an unstated assumption that there should be a basic blueprint of the mammalian brain, upon which evolution adds, modifies, and improves the plan and adapts novel solutions to help resolve challenging circumstances. The selection of a research subject is often guided by the premise that a specific feature of the subject can be related back to some human quality for translational medical relevance, to a basic generalizable plan of the nervous system, or to some remarkable specialization such as echo location. The immediate aim might be to identify in a simpler system how a particular process works, and the bigger picture might be to reveal brain specializations that evolved to optimize a process that ensures a species survival in a particular habitat. There is, rightly or wrongly, an assumption that the nervous system uses a fixed set of solutions to improve information processing and species survival. In the face of species specializations and even minor genetic variations in the same species, cognitive demands placed on communication, hunting, predator avoidance, and reproductive success could have consequences on brain structure and function yet to be determined. The fine details of how auditory circuits are conceptually and technically constructed are crucial for understanding the biology of hearing.

## Data availability statement

The original contributions presented in the study are included in the article/Supplementary material, further inquiries can be directed to the corresponding author.

## Ethics statement

This study was reviewed and approved by the Garvan Institute of Medical Research and St Vincent’s Hospital Animal Ethics Committee. This study was performed in strict accordance with the Australian Code for the Care and Use of Animals for Scientific Purposes (2013) and the ethical guidelines of the National Health and Medical Research Council (NHMRC) of Australia. All animals were handled according to Animal Ethics Committee protocols (Animal Research Authority: 19-33, 20-02, and 21-13).

## Author contributions

IW: Writing–review and editing, Writing–original draft, Visualization, Software, Resources, Project administration, Methodology, Investigation, Formal analysis, Data curation, Conceptualization. DR: Writing–review and editing, Writing–original draft, Validation, Supervision, Project administration, Funding acquisition, Conceptualization.

## Footnotes

1
https://www.jax.org

## References

[B1] Adams J. (1979). Ascending projections to the inferior colliculus. *J. Comp. Neurol.* 183 519–538. 10.1002/cne.901830305 759446

[B2] Adams J. C. (1981). Heavy metal intensification of DAB-based HRP reaction product. *J. Histochem. Chytochem.* 29:775. 10.1177/29.6.7252134 7252134

[B3] Altieri S. C. Zhao T. Jalabi W. Maricich S. M. (2014). Development of glycinergic innervation to the murine LSO and SPN in the presence and absence of the MNTB. *Front. Neural Circuits* 8:109. 10.3389/fncir.2014.00109 25309335 PMC4162373

[B4] Ansorge J. Wu C. Shore S. E. Krieger P. (2021). Audiotactile interactions in the mouse cochlear nucleus. *Sci. Rep.* 11:6887. 10.1038/s41598-021-86236-9 33767295 PMC7994829

[B5] Apfelbach R. Blanchard C. D. Blanchard R. J. Hayes R. A. McGregor I. S. (2005). The effects of predator odors in mammalian prey species: a review of field and laboratory studies. *Neurosci. Biobehav. Rev.* 29 1123–1144. 10.1016/j.neubiorev.2005.05.005 16085312

[B6] Avenant N. L. Smith V. R. (2003). The microenvironment of house mice on Marion Island (sub-Antarctic). *Polar Biol.* 26 129–141. 10.1007/s00300-002-0464-x

[B7] Banks M. I. Smith P. H. (1992). Intracellular recordings from Neurobiotin-labeled cells in brain slices of the rat medial nucleus of the trapezoid body. *J. Neurosci.* 12 2819–2837. 10.1523/JNEUROSCI.12-07-02819.1992 1351938 PMC6575844

[B8] Barker A. J. Koch Lewin G. R. Pyott S. J. (2021). Hearing and vocalizations in the naked mole-rat. *Adv. Exp. Med. Biol.* 1319 157–195. 10.1007/978-3-030-65943-1 34424516

[B9] Barnes-Davies M. Barker M. C. Osmani F. Forsythe I. D. (2004). Kv1 currents mediate a gradient of principal neuron excitability across the tonotopic axis in the rat lateral superior olive. *Eur. J. Neurosci.* 19 325–333. 10.1111/j.0953-816x.2003.03133.x 14725627

[B10] Barratt D. G. (1997). Home range size, habitat utilisation and movement patterns of suburban and farm cats *Felis catus*. *Ecography* 20 271–280. 10.1111/j.1600-0587.1997.tb00371.x

[B11] Begall S. Burda H. Schleich C. (2007). *Subterranean rodents: News from underground.* Berlin: Springer Science+Business Media.

[B12] Benichoux V. Ferber A. Hunt S. Hughes E. Tollin D. (2018). Across species “natural ablation” reveals the brainstem source of a noninvasive biomarker of binaural hearing. *J. Neurosci.* 38 8563–8573. 10.1523/jneurosci.1211-18.2018 30126974 PMC6170984

[B13] Berlin C. L. (1963). Hearing in mice via GSR audiometry. *J. Speech Hear. Res.* 6 359–368. 10.1044/jshr.0604.359 14071873

[B14] Berrebi A. S. Spirou G. A. (1998). PEP-19 immunoreactivity in the cochlear nucleus and superior olive of the cat. *Neuroscience* 83 535–554. 10.1016/s0306-4522(97)00407-7 9460761

[B15] Blaesse P. Ehrhardt S. Friauf E. Nothwang H.G. (2005). Developmental pattern of three vesicular glutamate transporters in the rat superior olivary complex. *Cell Tissue Res.* 320 33–50. 10.1007/s00441-004-1054-8 15714284

[B16] Bogaerts S. Clements J. D. Sullivan J. M. Oleskevich S. (2009). Automated threshold detection for auditory brainstem responses: Comparison with visual estimation in a stem cell transplantation study. *BMC Neurosci.* 10:104. 10.1186/1471-2202-10-104 19706195 PMC3224692

[B17] Bonthuis P. J. Cox K. H. Searcy B. T. Kumar P. Tobet S. Rissman E. F. (2010). Of mice and rats: Key species variations in the sexual differentiation of brain and behavior. *Front. Neuroendocrinol.* 31:341–358. 10.1016/j.yfrne.2010.05.001 20457175 PMC2910167

[B18] Boudreau J. C. Tsuchitani C. (1968). Binaural interaction in the cat superior olive s segment. *J. Neurophysiol.* 31 442–454. 10.1152/jn.1968.31.3.442 5687764

[B19] Boudreau J. C. Tsuchitani C. (1970). Cat superior olive S-segment cell discharge to tonal stimulation. *Contrib. Sens. Physiol.* 4 143–213. 10.1016/B978-0-12-151804-2.50011-5 4914058

[B20] Brunso-Bechtold J. K. Linville M. C. Henkel C. K. (1994). Terminal types on ipsilaterally and contralaterally projecting lateral superior olive cells. *Hear. Res.* 77 99–104. 10.1016/0378-5955(94)90257-7 7928743

[B21] Buffenstein R. Park T. Hanes M. Artwohl J. E. (2012). *Naked mole rat. The laboratory rabbit, guinea pig, hamster, and other rodents.* New York, NY: Elsevier, 1055–1074. 10.1016/b978-0-12-380920-9.00045-6

[B22] Cant N. B. (1984). The fine structure of the lateral superior olivary nucleus of the cat. *J. Comp. Neurol.* 227 63–77. 10.1002/cne.902270108 6470211

[B23] Cant N. B. Benson C. G. (2006). Wisteria floribunda lectin is associated with specific cell types in the ventral cochlear nucleus of the gerbil, *Meriones unguiculatus*. *Hear. Res.* 216–217 64–72. 10.1016/j.heares.2006.01.008 16497454

[B24] Cant N. B. Casseday J. H. (1986). Projections from the anteroventral cochlear nucleyus to the lateral and medial superior olivary nuclei. *J. Comp. Neurol.* 247 457–476. 10.1002/cne.902470406 3722446

[B25] Cloutman L. L. (2013). Interaction between dorsal and ventral processing streams: Where, when and how? *Brain Lang.* 127 251–263. 10.1016/j.bandl.2012.08.003 22968092

[B26] Darrow K. N. Maison S. F. Liberman M. C. (2006). Cochlear efferent feedback balances interaural sensitivity. *Nat. Neurosci.* 9 1474–1476. 10.1038/nn1807 17115038 PMC1806686

[B27] Davison A. Birks J. D. S. Griffiths H. I. Kitchener A. C. Biggins D. Butlin R. K. (1999). Hybridization and the phylogenetic relationship between polecats and domestic ferrets in Britain. *Biol. Conserv.* 87 155–161. 10.1016/S0006-3207(98)00067-6

[B28] Dewson J. H. (1967). Efferent Olivocochlear Bundle: Some relationships to noise masking and to stimulus attenuation. *J. Neurophysiol.* 30 817–832. 10.1152/jn.1967.30.4.817 6035692

[B29] Doucet J. R. Ryugo D. K. (2003). Axonal pathways to the lateral superior olive labeled with biotinylated dextran amine injections in the dorsal cochlear nucleus of rats. *J. Comp. Neurol.* 461 452–465. 10.1002/cne.10722 12746862

[B30] Driscoll C. A. Clutton-Brock J. Kitchener A. C. O’Brien S. J. (2009). The taming of the cat. *Sci. Am.* 30 68–75. PMC5790555 19485091

[B31] Dunbar R. I. M. (2009). Darwin and the ghost of phineas gage: Neuro-evolution and the social brain. *Cortex* 45 1119–1125. 10.1016/j.cortex.2009.05.005 19559409

[B32] Ehret G. Riecke S. (2001). Mice and humans perceive multiharmonic communication sounds in the same way. *Proc. Natl. Acad. Sci. U.S.A.* 99 479–482. 10.1073/pnas.012361999 11756654 PMC117585

[B33] Elliott L. (1978). Social behavior and foraging ecology of the Eastern Chipmunk (*Tamias striatus*) in the Adirondack mountains. *Smithsonian Contrib. Zool.* 265 1–107. 10.5479/si.00810282.265

[B34] Eybalin M. (1993). Neurotransmitters and neuromodulators of the mammalian cochlea. *Physiol. Rev.* 73 309–373. 10.1152/physrev.1993.73.2.309 8097330

[B35] Fech T. Calderón-Garcidueñas Kulesza R. J. Jr. (2017). Characterization of the superior olivary complex of *Canis lupus* domesticus. *Hear. Res.* 351 130–140. 10.1016/j.heares.2017.06.010 28633959

[B36] Finlayson P. G. Caspary D. M. (1991). Low-frequency neurons in the lateral superior olive exhibit phase-sensitive binaural inhibition. *J. Neurophysiol.* 65 598–605. 10.1152/jn.1991.65.3.598 2051197

[B37] Fisher M. F. Llewellyn G. C. (1978). The Mongolian gerbil: Natural history, care, and maintenance. *Am. Biol. Teach.* 40 557–560. 10.2307/4446413

[B38] Fontaine D. A. Davis D. B. (2016). Attention to background strain is essential for metabolic research: C57BL/6 and the international knockout mouse consortium. *Diabetes* 65 25–33. 10.2337/db15-0982 26696638 PMC4686949

[B39] Franken T. P. Joris P. X. Smith P. H. (2018). Principal cells of the brainstem’s interaural sound level detector are temporal differentiators rather than integrators. *Elife* 7:e33854. 10.7554/eLife.33854 29901438 PMC6063729

[B40] Fremeau R. T. Troyer M. D. Pahner I. Nygaard G. O. Tran C. H. Reimer R. J. (2001). The expression of vesicular glutamate transporters defines two classes of excitatory synapse. *Neuron* 31 247–260. 10.1016/s0896-6273(01)00344-0 11502256

[B41] Fremeau R. T. Voglmaier S. Seal R. P. Edwards R. H. (2004). VGLUTs define subsets of excitatory neurons and suggest novel roles for glutamate. *Trends Neurosci.* 27 98–103. 10.1016/j.tins.2003.11.005 15102489

[B42] Friauf E. Aragón C. Löhrke S. Westenfelder B. Zafra F. (1999). Developmental expression of the GlycineTransporter GLYT2 in the auditory system of rats suggests involvement in synapse maturation. *J. Comp. Neurol.* 412 17–37. 10.1002/(SICI)1096-9861(19990913)412:1<17::AID-CNE2<3.0.CO;2-E 10440707

[B43] Gessele N. Garcia-Pino E. Omerbašić D. Park T. J. Koch U. (2016). Structural changes and lack of HCN1 channels in the binaural auditory brainstem of the naked mole-rat (*Heterocephalus glaber*). *PLoS One* 11:e0146428. 10.1371/journal/pone.0146428 26760498 PMC4711988

[B44] Glendenning K. K. Mastereton R. B. (1983). Acoustic chiasm: Efferent projections of the lateral superior olive. *J. Neurosci.* 3 1521–1537. 10.1523/JNEUROSCI.03-08-01521.1983 6875655 PMC6564523

[B45] Glendenning K. K. Masterton R. B. (1998). Comparative morphometry of mammalian central auditory systems: Variation in nucleiu and form of the ascending system. *Brain Behav. Evol.* 51 59–89. 10.1159/000006530 9491274

[B46] Glendenning K. K. Baker B. N. Hutson K. A. Masterton R. B. (1992). Acoustic chiasm V: Inhibition and excitation in the ipsilateral and contralateral projections of LSO. *J. Comp. Neurol.* 319 100–122. 10.1002/cne.903190110 1317390

[B47] Gomeza J. Ohno K. Betz H. (2003). Glycine transporter isoforms in the mammalian central nervous system: Structures, functions and therapeutic promises. *Curr. Opin. Drug Discov. Dev.* 6 675–682. 14579517

[B48] Gómez-Álvarez M. Saldaña E. (2016). Different tonotopic regions of the lateral superior olive receive a similar combination of afferent inputs. *J. Comp. Neurol.* 524 2230–2250. 10.1002/cne.23942 26659473

[B49] Goupell M. J. Stakhovskaya O. A. (2018). Across-channel interaural-level-difference processing demonstrates frequency dependence. *J. Acoust. Soc. Am.* 143 645–658. 10.1121/1.5021552 29495743 PMC5798994

[B50] Grothe B. (1994). Interaction of excitation and inhibition in processing of pure tone and amplitude-modulated stimuli in the medial superior olive of the mustached bat. *J. Neurophysiol.* 71 706–721. 10.1152/jn.1994.71.2.706 8176433

[B51] Grothe B. (2000). The evolution of temporal processing in the medial superior olive, an auditory brainstem structure. *Prog. Neurobiol.* 61 581–610. 10.1016/s0301-0082(99)00068-4 10775798

[B52] Grothe B. Park T. J. (1995). Time can be traded for intensity in the lower auditory system. *Naturwissenschaften* 82 521–523. 10.1007/bf01134488 8544878

[B53] Guinan J. J. Norris B. E. Guinan S. S. (1972). Single auditory units in the superior olivary complex: II: Locations of unit categories and tonotopic organization. *Int. J. Neurosci.* 4 147–166. 10.3109/00207457209164756

[B54] Haragopal H. Mellott J. G. Dhar M. Kanel A. Mafi A. Tokar N. (2023). Tonotopic distribution and inferior colliculus projection pattern of inhibitory and excitatory cell types in the lateral superior olive of mice. *J. Comp. Neurol.* 531 1381–1388. 10.1002/cne.25515 37436768 PMC11571233

[B55] Harper N. S. McAlpine D. (2004). Optimal neural population coding of an auditory spatial cue. *Nature* 430 682–686. 10.1038/nature02768 15295602

[B56] Harper N. S. Scott B. H. Semple M. N. McAlpine D. (2014). The neural code for auditoryu space depends on sound frequency and head size in an optimal manner. *PLoS One* 9:e108154. 10.137/journal.pone.0108154 PMC4220907 25372405

[B57] Harris S. (1979). History, distribution, status and habitat requirements of the harvest mouse (*Micromys minutus*) in Britain. *Mammal. Rev.* 9 159–171. 10.1111/j.1365-2907.1979.tb00253.x

[B58] Hasan M. T. Friedrich R. W. Euler T. Larkum M. E. Giese G. (2004). Functional fluorescent Ca2+ indicator proteins in transgenic mice under TET control. *PLoS Biol.* 2:0763. 10.1371/journal.pbio.0020163 15208716 PMC423138

[B59] Hawrylycz M. Baldock R. A. Burger A. Hashikawa T. Johnson G. A. (2011). Digital atlasing and standardization in the mouse brain. *PLoS Comp. Biol.* 7:e1001065. 10.1371/journal.pcbi.1001065 21304938 PMC3033370

[B60] Hedreen J. C. (1998). What was wrong with the Abercrombie and empirical cell counting methods? A review. *Anat. Rec.* 250 373–380. 9517854 10.1002/(SICI)1097-0185(199803)250:3<373::AID-AR12>3.0.CO;2-L

[B61] Heffner R. S. Masterton R. B. (1990). “Sound localization in mammals: Brain-stem mechanisms,” in *Comparative perception*, Vol. 1 eds Berkley M. A. Stebbins W. C. (New York, NY: John Wiley & Sons), 285–314.

[B62] Helfert R. H. Schwartz I. R. (1986). Morphological evidence for the existence of multiple neuronal classes in the cat lateral superior olivary nucleus. *J. Comp. Neurol.* 244 533–549. 10.1002/cne.902440409 2420837

[B63] Helfert R. H. Schwartz I. R. (1987). Morphological features of five neuronal classes in the gerbil lateral superior olive. *Am. J. Anat.* 179 55–69. 10.1002/aja.1001790108 3618521

[B64] Helfert R. H. Bonneau J. M. Wenthold R. J. Altschuler R. A. (1989). GABA and glycine immunoreactivity in the guinea pig superior olivary complex. *Brain Res.* 501 269–286. 10.1016/0006-8993(89)90644-6 2819441

[B65] Helfert R. H. Juiz J. J. Bledsoe S. C. Bonneau J. M. Wenthold R. J. Altschuler R. A. (1992). Patterns of glutamate, glycine, and GABA immunolabeling in four synaptic terminal classes in the lateral superior olive of the guinea pig. *J. Comp. Neurol.* 323 305–325. 10.1002/cne.903230302 1360986

[B66] Henkel C. K. Brunso-Bechtold J. K. (1993). Laterality of superior olive projections to the inferior colliculus in adult and developing ferret. *J. Comp. Neurol.* 331 458–468. 10.1002/cne.903310403 8509504

[B67] Henry K. R. Chole R. A. (1980). Genotypic differences in behavioral, physiological and anatomical expressions of age-related hearing loss in the laboratory mouse. *Audiology* 19 369–383. 10.3109/00206098009070071 7436856

[B68] Heth G. Frankenberg E. Nevo E. (1986). Adaptive optimal sound for vocal communication in tunnels of a subterranean mammal (*Spalax ehrenbergi*). *Experientia* 42 1287–1289. 10.1007/BF01946426 3780955

[B69] Hikishima K. Komaki Y. Seki F. Ohnishi Y. Okano H. J. Okano H. (2017). In vivo microscopic voxel-based morphometry with a brain template to characterize strain-specific structures in the Mouse Brain. *Sci. Rep.* 7:85. 10.1038/s41598-017-00148-1 28273899 PMC5427914

[B70] Hind J. E. Goldberg J. M. Greenwood D. D. Rose J. E. (1963). Some discharge characteristics of single neurons in the inferior colliculus of the cat. II. Timing of the discharges and observations on binaural stimulation. *J. Neurophysiol.* 26 321–341. 10.1152/jn.1963.26.2.321 13954634

[B71] Humason G. L. (1979). *Animal tissue techniques*, 4th Edn. San Francisco, CA: W.H. Freeman and Company.

[B72] Irving R. Harrison J. (1967). The superior olivary complex and audition: A comparative study. *J. Comp. Neurol.* 130 77–86. 10.1002/cne.901300105 4962091

[B73] Ison J. R. Allen P. D. (2003). Low-frequency tone pips elicit exaggerated startle reflex in C57BL/6J mice with hearing loss. *J. Assoc. Res. Otolaryngol.* 4 495–504. 10.1007/s10162-002-3046-2 12784135 PMC3202743

[B74] Ito T. Oliver D. L. (2010). Origins of glutamatergic terminals in the inferior colliculus identified by retrograde transport and expression of VGLUT1 and VGLUT2 genes. *Front. Neuroanat.* 4:135. 10.3389/fnana.2010.00135 21048892 PMC2967334

[B75] Ito T. Bishop D. C. Oliver D. L. (2010). Expression of Glutamate and inhibitory amino acid vesicular transporters in the rodent auditory brainstem. *J. Comp. Neurol.* 519 316–340. 10.1002/cne.22521 21165977 PMC3092437

[B76] Jeffress L. A. (1948). A place theory of sound localization. *J. Comp. Physiol. Psychol.* 41 35–39. 10.1037/h0061495 18904764

[B77] Jenkins S. A. Simmons D. D. (2006). GABAergic neurons in the lateral superior olive of the hamster are distinguished by differential expression of gad isoforms during development. *Brain Res.* 1111 12–25. 10.1016/j.brainres.2006.06.067 16919247

[B78] Joris P. X. Yin T. C. T. (1998). Envelope coding in the lateral superior olive. III. Comparison with afferent pathways. *J. Neurophysiol.* 79 253–269. 10.1152/jn.1998.79.1.253 9425196

[B79] Kandler K. Kullmann P. Ene F. Kim G. (2002). Excitatory action of an immature glycinergic/GABAergic sound localization pathway. *Physiol. Behav.* 77 583–587. 10.1016/S0031-9384(02)00905-8 12527003

[B80] Kanold P. O. Young E. D. (2001). Proprioceptive information from the pinna provides somatosensory input to cat dorsal cochlear nucleus. *J. Neurosci.* 21 7848–7858. 10.1523/jneurosci.21-19-07848.2001 11567076 PMC6762891

[B81] Kanold P. O. Davis K. A. Young E. D. (2011). Somatosensory context alters auditory responses in the cochlear nucleus. *J. Neurophysiol.* 105 1063–1070. 10.1152/jn.00807.2010 21178001 PMC3295206

[B82] Kelly J. B. Liscum A. van Adel B. Ito M. (1998). Projections from the superior olive and lateral lemniscus to tonotopic regions of the rat’s inferior colliculus. *Hear. Res.* 116 43–54. 10.1016/S0378-5955(97)00195-0 9508027

[B83] Klug A. Park T. J. Pollak G. D. (1995). Glycine and GABA influence binaural processing in the inferior colliculus of the mustache bat. *J. Neurophysiol.* 74 1701–1713. 10.1152/jn.1995.74.4.1701 8989406

[B84] Koch U. Sanes D. H. (1998). Afferent regulation of glycine receptor distribution in the gerbil LSO. *Microsc. Res. Tech.* 41 263–269. 10.1002/(sici)1097-0029(19980501)41:3<263::aid-jemt9>3.0.co;2-u 9605343

[B85] Konishi M. (2000). Study of sound localization by owls and its relevance to humans. *Comp. Biochem. Physiol. A Mol. Integr. Physiol.* 126 459–469. 10.1016/s1095-6433(00)00232-4 10989338

[B86] Kulesza R. J. Jr. (2008). Cytoarchitecture of the human superior olivary complex: Nuclei of the trapezoid body and posterior tier. *Hear. Res.* 241 52–63. 10.1016/j.heares.2008.04.010 18547760

[B87] Kulesza R. J. Jr. (2014). Characterization of human auditory brainstem circuits by calcium-binding protein immunohistochemistry. *Neuroscience* 258 318–331. 10.1016/j.neuroscience.2013.11.035 24291726

[B88] Kuwabara N. Zook J. M. (1991). Classification of the principal cells of the medial nucleus of the trapezoid body. *J. Comp. Neurol.* 314 707–720. 10.1002/cne.903140406 1816272

[B89] Kuwada S. Yin T. C. Buunen T. J. Wickesberg R. E. (1984). Binaural interaction in low-frequency neurons in inferior colliculus of the cat. IV. Comparison of monaural and binaural response properties. *J. Neurophysiol.* 51 1306–1325. 10.1152/jn.1984.51.6.1306 6737032

[B90] Lanciego J. L. Wouterlood F. G. (2020). Neuroanatomical tract-tracing techniques that did go viral. *Brain Struct. Funct.* 225 1193–1224. 10.1007/s00429-020-02041-6 32062721 PMC7271020

[B91] LaVail J. H. Winston K. R. Tish A. (1973). A method based on retrograde intraaxonal transport of protein for identification of cell bodies of origin of axons terminating within the CNS. *Brain Res.* 58 470–477. 10.1016/0006-8993(73)90016-4 4127874

[B92] Li S.-H. Zhang C.-K. Qiao Y. Ge S.-N. Zhang T. Li J.-L. (2020). Coexpression of VGLUT1 and VGLUT2 in precerebellar neurons in the lateral reticular nucleus of the rat. *Brain Res. Bull.* 162 94–106. 10.1016/j.brainresbull.2020.06.008 32562720

[B93] Liberman M. C. (1980). Efferent synapses in the inner hair cell area of the cat cochlea: An electron microscopic study of serial sections. *Hear. Res.* 3 189–204. 10.1016/0378-5955(80)90046-5 7440423

[B94] Liu Y.-J. Li L.-F. Zhang Y.-H. Guo H.-F. Xia M. Zhang M.-W. (2017). Chronic co-species housing mice and rats increased the competitiveness of male mice. *Chem. Sens.* 42 247–257. 10.1093/chemse/bjw164 28073837

[B95] Loftus W. C. Bishop D. C. Oliver D. L. (2010). Differential patterns pof inputs create functional zones in central nucleus of inferior colliculus. *J. Neurosci.* 30 13396–133408. 10.1523/JNEUROSCI.0338-10.2010 20926666 PMC2966845

[B96] Loftus W. C. Bishop D. C. Saint Marie R. L. Oliver D. L. (2004). Organization of binaural excitatory and inhibitory inputs to the inferior colliculus from the superior olive. *J. Comp. Neurol.* 472 330–344. 10.1002/cne.20070 15065128

[B97] McKay J. (2012). *New complete guide to ferrets.* Stroud: Quiller Publishing Ltd.

[B98] Maison S. F. Adams J. C. Liberman M. C. (2002). Olivocochlear innervation in the mouse: Immunocytochemical maps, crossed versus uncrossed contributions, and transmitter colocalization. *J. Comp. Neurol.* 455 406–416. 10.1002/cne.10490 12483691 PMC1805785

[B99] Malmierca M. S. Ryugo D. K. (2011). “Descending connections of auditory cortex to the midbrain and brain stem,” in *The auditory cortex*, eds Winer J. A. Schriener C. E. (New York, NY: Springer), 189–208. 10.1007/978-1-4419-0074-6_9

[B100] Marshall F. (2020). Cats as predators and early domesticates in ancient human landscapes. *Proc. Nat. Acad. Sci. U.S.A.* 117 18154–18156. 10.1073/pnas.2011993117 32690714 PMC7414175

[B101] Martinez-Lozada Z. Ortega A. (2023). Milestone review: Excitatory amino acid transporters – beyond their expected function. *J. Neurochem.* 165 457–466. 10.1111/jnc.15809 36920226

[B102] Masterton B. Heffner H. Ravizza R. (1969). The evolution of human hearing. *J. Acoust. Soc. Am.* 45 966–985. 10.1121/1.1911574 5791616

[B103] Masterton R. B. Diamond I. T. Harrison J. M. Beecher M. D. (1967). Medial superior olive and sound localization. *Science* 155 1696–1697. 10.1126/science.155.3770.1696-a 17759539

[B104] Mellott J. G. Dhar M. Mafi A. Tokar N. Winters B. D. (2021). Tonotopic distribution and inferior colliculus projection pattern of inhibitory and excitatory cell types in the lateral superior olive of Mongolian gerbils. *J. Comp. Neurol.* 530 506–517. 10.1002/cne.25226 34338321 PMC8716415

[B105] Mesulam M. M. (1978). Tetramethyl benzidine for horseradish peroxidase neurohistochemistry: A non-carcinogenic blue reaction product with superior sensitivity for visualizing neural afferents and efferents. *J. Histochem. Cytochem.* 26 106–117. 10.1177/26.2.24068 24068

[B106] Milinkeviciute G. Muniak M. A. Ryugo D. K. (2017). Descending projections from the inferior colliculus to the dorsal cochlear nucleus are excitatory. *J. Comp. Neurol.* 525 773–793. 10.1002/cne.24095 27513294

[B107] Miller J. (1996). The domestic cat: Perspective on the nature and diversity of cats. *J. Am. Vet. Med. Assoc.* 208 498–502. 10.2460/javma.1996.208.04.498a 8603894

[B108] Moore D. R. Russell F. A. Cathcart N. C. (1995). Lateral superior olive projections to the inferior colliculus in normal and unilaterally deafened ferrets. *J. Comp. Neurol.* 357 204–216. 10.1002/cne.903570203 7545189

[B109] Moore J. K. Moore R. Y. (1971). A comparative study of the superior olivary complex in the primate brain. *Folia Primatol.* 16 35–51. 10.1159/000155390 4111530

[B110] Mosieff A. Konishi M. (1981). Neuronal and behavioral sensitivity to binaural time differences in the owl. *J. Neurosci.* 1 40–48. 10.1523/JNEUROSSCI.01-01-00040.1981 7346557 PMC6564159

[B111] Muniak M. A. Ayeni F. E. Ryugo D. K. (2018). Hidden hearing loss and endbulbs of Held: Evidence for central pathology before detection of ABR threshold increases. *Hear. Res.* 364 104–117. 10.1016/j.heares.2018.03.021 29598838

[B112] Nabekura J. Katsurabayashi S. Kakazu Y. Shibata S. Matsubara A. Jinno S. (2003). Developmental switch from GABA to glycine release in single central synaptic terminals. *Nat. Neurosci.* 7 17–23. 10.1038/nn1170 14699415

[B113] Nerlich J. Rübsamen R. Milenkovic I. (2017). Developmental shift of inhibitory transmitter content at a central auditory synapse. *Front. Cell. Neurosci.* 11:211. 10.3389/fncel.2017.00211 28769768 PMC5516124

[B114] Netser S. Meyer A. Magalnik H. Zylbertal A. de la Zerda S. H. Briller M. (2020). Distinct dynamics of social motivation drive differential social behavior in laboratory rat and mouse strains. *Nat. Commun.* 11:5908. 10.1038/s41467-020-19569-0 33219219 PMC7679456

[B115] Ngodup T. Romero G. E. Trussell L. O. (2020). Identification of an inhibitory neuron subtype, the L-stellate cell of the cochlear nucleus. *eLife* 9:e54350. 10.7554/eLife.54350 33141020 PMC7744103

[B116] Niu X. Bogdanovic N. Canlon B. (2004). The distribution and the modulation of tyrosine hydroxylase immunoreactivity in the lateral olivocochlear system of the guinea-pig. *Neuroscience* 125 725–733. 10.1016/j.neuroscience2004.02.023 15099686

[B117] Nordeen K. W. Killackey H. P. Kitzes L. M. (1983). Ascending auditory projections to the inferior colliculus in the adult gerbil, *Meriones unguiculatus*. *J. Comp. Neurol.* 214 131–143. 10.1002/cne.902140203 6841681

[B118] Ohlemiller K. K. Jones S. M. Johnson K. R. (2016). Application of mouse models to research in hearing and balance. *J. Assoc. Res. Otolaryngol.* 17:493e523. 10.1007/s10162-016-0589-1 27752925 PMC5112220

[B119] Okanoya K. Yosida S. Barone C. M. Applegate D. T. Brittan-Powell E. F. Dooling R. J. (2018). Auditory-vocal coupling in the naked mole-rat, a mammal with poor auditory thresholds. *J. Comp. Physiol. A Neuroethol. Sens. Neural Behav. Physiol.* 204 905–914. 10.1007/s00359-018-1287-8 30232547 PMC6208660

[B120] Oliver D. L. (2000). Ascending efferent projections of the superior olivary complex. *Microsc. Res. Tech.* 51 355–363. 10.1002/1097-0029(20001115)51:4<355::AID-JEMT5>3.0.CO;2-J 11071719

[B121] Ollo C. Schwartz I. R. (1979). The superior olivary complex in C57BL/6 mice. *Am. J. Anat.* 155 349–373. 10.1002/aja.1001550306 474450

[B122] Ono M. Ito T. (2015). Functional organization of the mammalian auditory midbrain. *J. Physiol. Sci.* 65 499–506. 10.1007/s12576-015-0394-3 26362672 PMC10718034

[B123] Ono M. Bishop D. C. Oliver D. L. (2020). Neuronal sensitivity to the interaural time difference of the sound envelope in the mouse inferior colliculus. *Hear. Res.* 385:107844. 10.1016/j.heares.2019.107844 31759235 PMC6933070

[B124] Ouagazzal A.-M. Reiss D. Romand R. (2006). Effeccts of age-related hearing loss on startle reflex and prepulse inhibition in mice on pure and mixed C57BL and 129 genetic background. *Behav. Brain Res.* 172 307–315. 10.1016/j.bbr.2006.05.018 16814879

[B125] Park T. J. Buffenstsein R. (2012). *Digging the underground life. The Scientist.**

[B126] Park T. J. Klug A. Holinstat M. Grothe B. (2004). Interaural level difference processing in the lateral superior olive and the inferior colliculus. *J. Neurophysiol*. 92, 289–301. 10.1152/jn.00961.2003 15056693

[B127] Paxinos G. Franklin B. J. (2008). *The mouse brain in stereotaxic coordinates*, 3rd Edn. Sydney, NSW: Elsevier.

[B128] Qiu L. Zhang B. Gao Z. (2022). Lighting up neural circuits by viral tracing. *Neurosci. Bull.* 38 1383–1396. 10.1007/s12264-022-00860-7 35578093 PMC9672192

[B129] Rauschecker J. P. (2018). Where, when, and how: Are they all sensorimotor? Towards a unified view of the dorsal pathway in vision and audition. *Cortex* 98 262–268. 10.1016/j.cortex.2017.10.020 29183630 PMC5771843

[B130] Reiner A. Veenman C. L. Medina L. Jiao Y. Del Mar N. Honig M. G. (2000). Pathway tracing using biotinylated dextran amines. *J. Neurosci Methods* 15 23–37. 10.1016/s0165-0270(00)00293-4 11074093

[B131] Reiss L. A. Young E. D. (2005). Spectral edge sensitivity in neural circuits of the dorsal cochlear nucleus. *J. Neurosci.* 25 3680–3691. 10.1523/JNEUROSCI.4963-04.2005 15814799 PMC6725373

[B132] Rice J. J. May B. J. Spirou G. A. Young E. D. (1992). Pinna-based spectral cues for sound localization in cat. *Hear. Res.* 58 132–152. 10.1016/0378-5955(92)90123-5 1568936

[B133] Rietzel H. J. Friauf E. (1998). Neuron types in the rat lateral superior olive and developmental changes in the complexity of their dendritic arbors. *J. Comp. Neurol.* 390 20–40. 10.1002/(SICI)1096-9861(19980105)390:1<20::AID-CNE3>3.0.CO;2-S 9456173

[B134] Romanski L. M. Tian B. Fritz J. Mishkin M. Goldman-Rakic P. S. Rauschecker J. P. (1999). Dual streams of auditory afferents target multiple domains in the primate prefrontal cortex. *Nat. Neurosci.* 2 1131–1136. 10.1038/16056 10570492 PMC2778291

[B135] Ross L. S. Pollak G. D. Zook J. M. (1988). Origin of ascending projections to an isofrequency region of the mustache bat’s inferior colliculus. *J. Comp. Neurol.* 270 488–505. 10.1002/cne.902700403 2836478

[B136] Rossi A. C. (1975). The “mouse-killing” rat: Ethological discussion on an experimental model of aggression. *Pharmacol. Res. Commun.* 7 199–216. 10.1016/0031-6989(75)90020-x 1170577

[B137] Ryugo D. K. Fekete D. M. (1982). Morphology of primary axosomatic endings in the anteroventral cochlear nucleus of the cat: A study of the endbulbs of Held. *J. Comp. Neurol.* 210 239–257. 10.1002/cne.902100304 7142440

[B138] Ryugo D. K. Milinkeviciute G. (2023). Differential projections from the cochlear nucleus to the inferior colliculus in the mouse. *Front. Neural Circuits* 17:1229746. 10.3389/fncir.2023.1229746 37554670 PMC10405501

[B139] Ryugo D. K. Haenggeli C.-A. Doucet J. R. (2003). Multimodal inputs to the granule cell domain of the cochlear nucleus. *Exp. Brain Res.* 153 477–485. 10.1007/s00221-003-1605-3 13680048

[B140] Ryugo D. K. Popper A. N. Fay R. R. (2011). *Auditory and vestibular efferents.* New York, NY: Springer-Verlag.

[B141] Saint Marie R. L. S. Ostapoff E.-M. Morest D. K. Wenthold R. J. (1989). Glycine-immunoreactive projection of the cat lateral superior olive: Possible role in midbrain ear dominance. *J. Comp. Neurol.* 279 382–396. 10.1002/cne902790305 2918077

[B142] Saint Marie R. L. Baker R. A. (1990). Neurotransmitter-specific uptake and retrograde transport of [3H]glycine from the inferior colliculus by ipsilateral projections of the superior olivary complex and nuclei of the lateral lemniscus. *Brain Res.* 524 244–253. 10.1016/0006-8993(90)90698-B 1705464

[B143] Schaeffer D. F. Reuss M. H. Riemann R. Reuss S. (2003). A nitrergic projection from the superior olivary complex to the inferior colliculus of the rat. *Hear. Res.* 183 67–72. 10.1016/s0378-5955(03)00219-3 13679139

[B144] Scheibel M. E. Scheibel A. B. (1974). Neuropil organization in the superior olive of the cat. *Exp. Neurol.* 43 339–348. 10.1016/0014-4886(74)90175-7 4826971

[B145] Scheibler E. Liu W. Weinandy R. Gattermann R. (2006). Burrow systems of the Mongolian gerbil (*Meriones unguiculatus* Milne Edwards, 1867). *Mammal. Biol.* 71 178–182. 10.1016/j.mambio.2005.11.007

[B146] Schweizer H. (1981). The connections of the inferior colliculus and the organization of the brainstem auditory system in the greater horseshoe bat (*Rhinolophus ferrumequinum*). *J. Comp. Neurol.* 201 25–49. 10.1002/cne902010104 7276250

[B147] Sergeyenko Y. Lall K. Liberman M. C. Kujawa S. G. (2013). Age-related cochlear synaptopathy: An early-onset contributor to auditory functional decline. *J. Neurosci.* 33 13686–13694. 10.1523/JNEUROSCI.1783-13.2013 23966690 PMC3755715

[B148] Smith J. C. (2019). A review of strain and sex differences in response to pain and analgesia in mice. *Comp. Med.* 69 490–500. 10.30802/AALAS-CM-19-000066 31822324 PMC6935701

[B149] Stevens S. S. Newman E. B. (1936). The localization of actual sources of sound. *Am. J. Psychol.* 48:297. 10.2307/1415748

[B150] Storer T. I. (1948). Control of rats and mice. California agricultural extension service. *Circular* 138:142. 10.5962/bhl.title.53398 33311142

[B151] Storm-Mathisen J. Leknes A. K. Bore A. T. Vaaland J. L. Edminson P. Haug F.-M. (1983). First visualization of glutamate and GABA in neurones by immunocytochemistry. *Nature* 301 517–520. 10.1038/301517a0 6130475

[B152] Suthakar K. (2017). *Changes in the descending auditory system in hearing loss: Focus on auditory efferents*. Ph.D. thesis. Sydney, NSW: University of New South Wales.

[B153] Taber E. (1961). The cytoarchitecture of the brain stem of the cat. I. Brain stem nuclei of cat. *J. Comp. Neurol.* 116 27–69. 10.1002/cne.901160104 13774738

[B154] Taberner A. M. Liberman M. C. (2005). Response properties of single auditory nerve fibers in the mouse. *J. Neurophysiol.* 93:557e569. 10.1152/jn00574.2004 15456804

[B155] Tollin D. J. Yin T. C. (2002). The coding of spatial location by single units in the lateral superior olive of the cat. I. Spatial receptive fields in azimuth. *J. Neurosci.* 22 1454–1467. 10.1523/jneurosci.22-04-01454.2002 11850472 PMC6757576

[B156] Tollin D. J. Yin T. C. (2005). Interaural phase and level difference sensitivity in low-frequency neurons in the lateral superior olive. *J. Neurosci.* 25, 10648–10657. 10.1523/jneurosci.1609-05.2005 16291937 PMC1449742

[B157] Traweger D. Travnitzky R. Moser C. Walzer C. Bernatzky G. (2006). Habitat preferences and distribution of the brown rat (rattus Norvegicus Berk.) in the city of Salzburg (Austria): Implications for an urban rat management. *J. Pest Sci.* 79 113–125. 10.1007/s10340-006-0123-z

[B158] Tsuchitani C. Boudreau J. C. (1966). Single unit analysis of cat superior olive s segment with tonal stimuli. *J. Neurophysiol.* 29 684–697. 10.1152/jn.1966.29.4.684 5966430

[B159] Tsuchitani C. Boudreau J. C. (1967). Encoding of stimulus frequency and intensity by cat superior olive S-segment cells. *J. Acoust. Soc. Am.* 42 794–805. 10.1121/1.1910651 6075565

[B160] Vetter D. E. Adams J. C. Mugnaini E. (1991). Chemically distinct rat olivocochlear neurons. *Synapse* 7 21–43. 10.1002/syn.890070104 1706537

[B161] Vetter D. E. Mugnaini E. (1992). Distribution and dendritic features of three groups of rat olivocochlear neurons. *Anat. Embryol.* 185 1–16. 10.1007/BF00213596 1736680

[B162] Vorhies C. T. Taylor W. P. (2015). *Life history of the kangaroo rat: Dipodomys spectabilis spectabilis merriam.* Boulder, CO: Palala Press.

[B163] Warr W. B. (1975). Olivocochlear and vestibular efferent neurons of the feline brain stem: Their location, morphology and number determined by retrograde axonal transport and acetylcholinesterase histochemistry. *J. Comp. Neurol.* 161 159–181. 10.1002/cne.901610203 47866

[B164] Weber J. N. Peterson B. K. Hoekstra H. E. (2013). Discrete genetic modules are responsible for complex burrow evolution in Peromyscus mice. *Nature* 493 402–405. 10.1038/nature11816 23325221

[B165] Wenthold R. J. Huie D. Altschuler R. A. Reeks K. A. (1986). Glycine immunoreactivity localized in the cochlear nucleus and superior olivary complex. *Neuroscience* 22 897–912. 10.1016/0306-4522(87)92968-x 3683855

[B166] Willard F. H. Martin G. F. (1984). Collateral innervation of the inferior colliculus in the North American opossum: A study using fluorescent markers in a double-labeling paradigm. *Brain Res.* 303 171–182. 10.1016/0006-8993(84)90225-7 6733522

[B167] Williams I. R. Filimontseva A. Connelly C. J. Ryugo D. K. (2022). The lateral superior olive in the mouse: Two systems of projecting neurons. *Front. Neural Circuits* 16:1038500. 10.37247/pans2ed.2.23.13 PMC9630946 36338332

[B168] Willott J. F. Turner J. T. (1999). Prolonged exposure to an augmented acoustic environment ameliorates age-related auditory changes in C59BL/6J and DB A/2J mice. *Hear. Res.* 135 78–88. 10.1016/s0378-5955(99)00094-5 10491957

[B169] Willott J. F. Pankow D. Hunter K. P. Kordyban M. (1985). Projections from the anterior ventral cochlear nucleus to the central nucleus of the inferior colliculus iin young and aging C57BL/6 mice. *J. Comp. Neurol.* 238 545–551. 10.1002/cne.902370410 3840181

[B170] Wright D. D. Ryugo D. K. (1996). Mossy fiber projections from the cuneate nucleus to the cochlear nucleus in the rat. *J. Comp. Neurol.* 365 159–172. 10.1002/(sici)1096-9861(19960129)365:1&lt159::aid-cne12>3.0.co;2-l 8821448

[B171] Wu C. Shore S. E. (2018). Multisensory activation of ventral cochlear nucleus d-stellate cells modulates dorsal cochlear nucleus principal cell spatial coding. *J. Physiol.* 596 4537–4548. 10.1113/jp276280 30074618 PMC6138285

[B172] Wu J. S. Yi E. Manca M. Javaid H. Lauer A. M. Glowatzki E. (2020). Sound exposure dynamically induces dopamine synthesis in cholinergic LOC efferents for feedback to auditory nerve fibers. *Elife* 9:e52419. 10.7554/eLife.52419 31975688 PMC7043886

[B173] Yin T. C. T. Smith P. H. Joris P. X. (2019). Neural mechanisms of binaural processing in the auditory brainstem. *Comp. Physiol.* 9 1503–1575. 10.1002/cphy.c180036 31688966

[B174] Zeilhofer H. U. Studler B. Arabadzisz D. Schweizer C. Ahmadi S. Layh B. (2005). Glycinergic neurons expressing enhanced green fluorescent protein in bacterial artificial chromosome transgenic mice. *J. Comp. Neurol.* 482 123–141. 10.1002/cne.20349 15611994

[B175] Zheng Q. Y. Johnson K. R. Erway L. C. (1999). Assessment of hearing in 80 inbred strains of mice by ABR threshold analyses. *Hear. Res.* 130 94–107. 10.1016/s0378-5955(99)00003-9 10320101 PMC2855304

[B176] Zhu X. Vasilyeve O. N. Kim S. Jacobson M. Romney J. (2007). Auditory efferent feedback system deficits precede age-related hearing loss: Contralateral suppression of otoacoustic emissions in mice. *J. Comp. Neurol.* 503 593–604. 10.1002/cne.21402 17559088

