# Supplementary material for: Bilateral and symmetric glycinergic and glutamatergic projections from the LSO to the IC in the CBA/CaH mouse
Source: Front Neural Circuits. 2024 Aug 9;18:1430598. doi: 10.3389/fncir.2024.1430598 (PMC11341401; doi:10.3389/fncir.2024.1430598)
Supplement: Supplementary file 1 [file Table_1.docx]

| **Williams et al., 2022 Williams & Ryugo 2024** | **Cell Counts** | | | | |
| --- | --- | --- | --- | --- | --- |
| **CBA/CaH Mouse** | **Ipsilateral** | **Contralateral** | | **Total** | |
| Cresyl Violet | 2277 | 2785 | | 5062 | |
| LOCs - ChAT |  |  | | 362 ± 25.4 | |
| LOCs - AChE |  |  | | 357.8 ± 18.6 | |
| IC projecting neurons | 704 ± 201.6 | 701 ± 152.2 | | 1405 ± 345.8 | |
| Non-IC-projecting glycine cells | 239.3 ± 61.6 | 255.7 ± 48.8 | | 495 ± 97.7 | |
| Non-IC-projecting glutamate cells | 336.4 ± 80.9 | 335.8 ± 98.1 | | 672.2 ± 178.2 | |
| LOCs + IC projecting neurons | 1423.8 | 1313.5 | | 2737.3 | |
| **Others** | **Label** | **Ipsilateral** | **Contralateral** | | **Total** |
| *Campbell & Hensen (1988)* - **mouse** | HRP | 311 | 0 | | 311 |
| *Brown & Levine (2008)* **mouse** | ChAT | 214.9 | 0 | | 214.9 |
| *Ryan et al., (1987)* **gerbil** | LOCs |  |  | | ~500 |
|  | Total Cells |  |  |  | 2374 |
| *Helfert & Schwartz (1986)* - **cat** | All LSO cells labeled by Golgi staining |  |  | | 2517 |
| *Irving & Harrison (1967)* **mouse** | Total Cells |  |  | | 1190 |
| *Harrison & Feldman (1970)* **mouse** and **chinchilla** | Total cells - mouse |  |  | | 1190 |
|  | Total cells - chinchilla |  |  |  | 3320 |
| *Moore & Moore (1971)* **cat, gibbon, & human** | Total cells – cat |  |  | | 6533 |
|  | Total cells – gibbon |  |  |  | 2045 |
|  | Total cells – human |  |  |  | 2369 |
| *Haragopal et al.,* (2023) **C57 mouse** | No counts provided, %s only |  |  | |  |
| *Mellott et al., (2022)* **gerbil** | Glycine neurons | 562 | 26 | | 588 |
|  | Glutamate neurons | 818 | 1808 | | 2626 |
|  | Unlabeled neurons | 296 | 226 | | 622 |

**Supplementary Table 1. Comparison of LSO counts in our study to the literature.**
