# Supplementary material for: Bilateral and symmetric glycinergic and glutamatergic projections from the LSO to the IC in the CBA/CaH mouse
Source: Front Neural Circuits. 2024 Aug 9;18:1430598. doi: 10.3389/fncir.2024.1430598 (PMC11341401; doi:10.3389/fncir.2024.1430598)
Supplement: Supplementary file 2 [file Data_Sheet_1.docx]

**Supplementary Figure Legends**

**Supplementary Figure 1. Reconstructed injection sites in the IC of mice from representative members of each immunostaining set: GlyT2 and vLGUT2.** The injection sites are presented as border contours from a middle section of the IC. The FG injection parameters were the same for each group, but their final sizes as recovered histologically are variable. The vGLUT2 group tended to have larger injection sites with corresponding larger numbers of retrogradely labelled neurons. Scale bar equals 1mm.

**Supplementary Figure 2. GlyT2 labelled MNTB.** It is well established that neurons housed in the MNTB are glycinergic and label with glycinergic staining. Neurons of the MNTB were labelled using GlyT2 staining techniques and used as a positive control for LSO labelling (blue fluorescence). The cytoplasm of neurons in the MNTB strongly label with GlyT2; this labelling feature is also observed in LSO neurons. Scale bar equals 100 µm.

**Supplementary Figure 3. Cresyl violet (CV) staining, cell counts, and neuronal mapping.** 60µm-thick brainstem sections were stained by CV and neurons residing in the LSO were counted and mapped. **(A)** Counting criteria for neurons included in this study: principal neurons with visible nucleus, principal neurons with no visible nucleus, small neurons with visible nucleus, and small neurons with no visible nucleus. **(B)** Five photomicrographs of alternate CV-stained frontal sections are paired with their corresponding drawings through the left LSO (10 sections in total for the LSO). **(C)** Five photomicrographs of alternate CV-stained frontal sections are paired with their corresponding drawings through the right LSO. Cell counts are doubled to account for the skipped intervening sections: left LSO, n=5,570 cells; right LSO, n=4,554 cells; no correction factor was applied.

**Supplementary Figure 4.** **Criteria for including principal neurons in LSO morphometric analyses.**  (A) Neurons (yellow) labelled by FluoroGold injected into the IC. (B) Neurons immunostained by GlyT2. (C) Neurons immunostained by vGLUT2. These neurons had sharp somatic boundaries and a nucleus observable by focussing through the cell body. They were drawn, counted, and measured using microscopic criteria.

**Supplementary Figure 5. Large GlyT2 and vGLUT2 neurons in the LSO were labelled using immunohistochemistry.** (**A,** column 1**)** Low magnification micrographs with boxed areas showing the location of large, GlyT2-stained neurons. (**A,** column 2). High magnification photomicrograph of the boxed area capturing GlyT2-labelled neurons that are distinctly larger than other LSO neurons. (**B,** column 3) Low magnification micrographs with boxed areas showing the location of large vGLUT2-stained neurons. (**B,** column 4) High magnification examples of large, vGLUT2-labelled neurons from the boxed area.

**Supplementary Figure 6.**  **Cell size and shape measurements.** Labelled LSO neurons were drawn from high magnification photomicrographs and the somatic silhouette area (sq. µm) and shape (scale between 0-1, where 1 indicates a circle) determined using *FIJI* (ImageJ software). Cell area and shape were graphed: **(A)** single-labelled GlyT2 neurons; **(B)** GlyT2-labelled neurons projecting to the IC; **(C)** single-labelled vGLUT2 neurons; **(D)** vGLUT2-labelled neurons projecting to the IC; **(E)** IC-projecting neurons not immunostaining for either GlyT2 or vGLUT2; and **(F)** all cells. The ‘small’ GlyT2 neurons that do not project to the IC are disstinct; there were no small vGLUT2 neurons. The main cluster of neurons exhibit cell body area of around 100 µm^2^ and a roundness score of 0.6, which is consistent for LSO principal cells having a fusiform shape. The small GlyT2 somata are speculated as representing inhibitory local circuit neurons.

**Supplementary Figure 7. Schematic diagrams of burrow architecture for some common rodents.** Burrowing animals have underground tunnels of variable complexity, with more elaborate burrows reported for gerbils, chipmunks, and naked mole rat (Elliot, 1978; Scheibler et al., 2006; Park and Buffenstein, 2012). Rats and mice use relatively basic burrows featuring two-three openings and an expanded chamber for feeding and nesting (Storer, 1948; Weber et al., 2013). In contrast, the naked mole rat constructs an elaborate system of burrows, up to 3m in depth, 6km in length, and spread out across 6 football fields. Burrows limit the transmission of high frequency sounds and essentially minimize the need to process lateralized sounds. Audiograms, natural history, and relative position along the food chain will influence sound processing mechanisms.

**Supplementary Figure 8. Audiograms of nine different species.** Audiograms of the cat, ferret, gerbil, guinea pig, human, mouse, rat and naked mole rat show the variations in audiograms across a sample of mammals used for hearing research. These audiograms highlight the poor low frequency hearing and expanded high frequency hearing of the mouse (adapted from Fay, 1988).
