# Supplementary figures and images for: Bilateral and symmetric glycinergic and glutamatergic projections from the LSO to the IC in the CBA/CaH mouse
Source: Front Neural Circuits. 2024 Aug 9;18:1430598. doi: 10.3389/fncir.2024.1430598 (PMC11341401; doi:10.3389/fncir.2024.1430598)

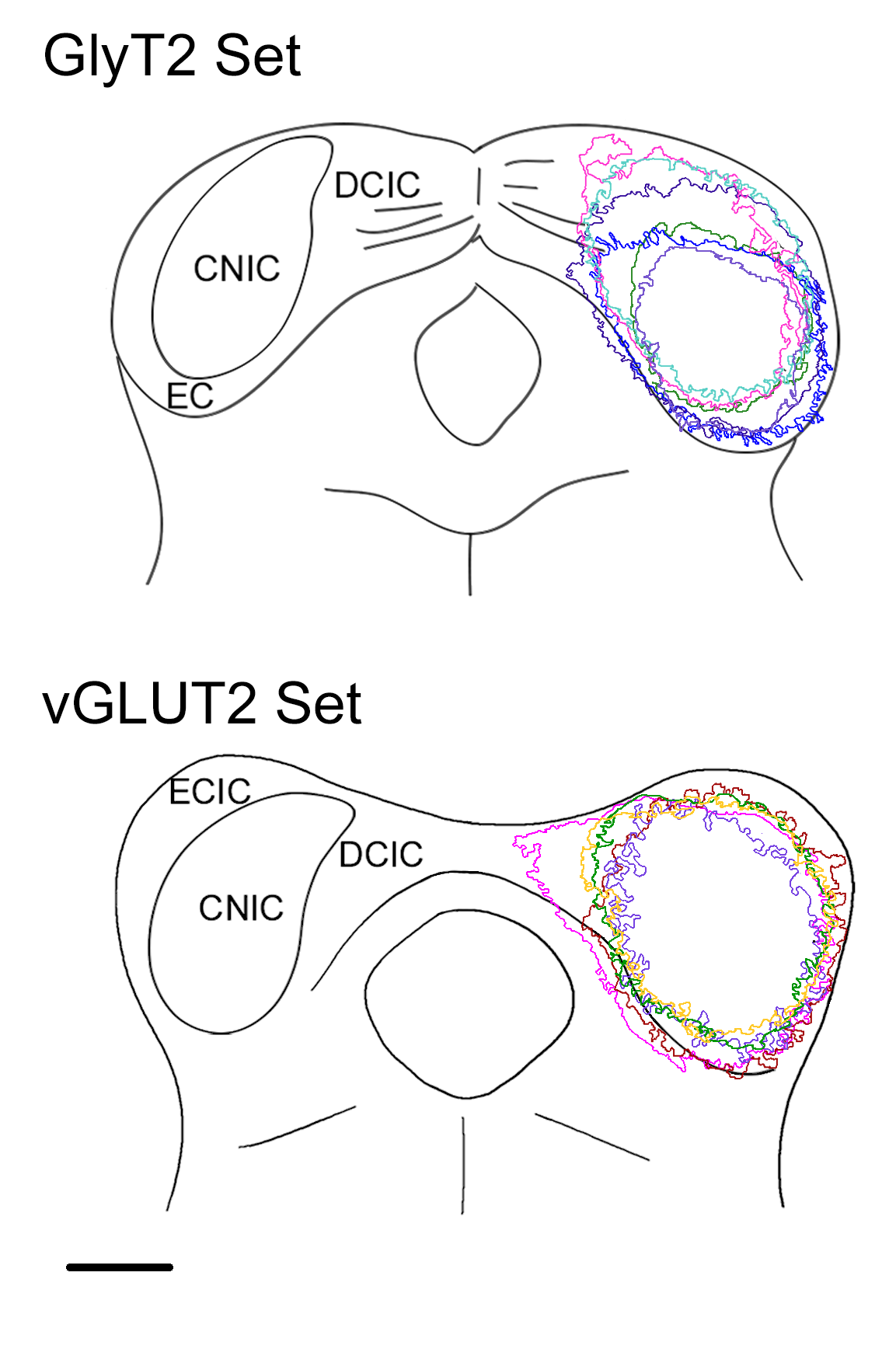

Supplement: Supplementary file 3 [file Image_1.tif]

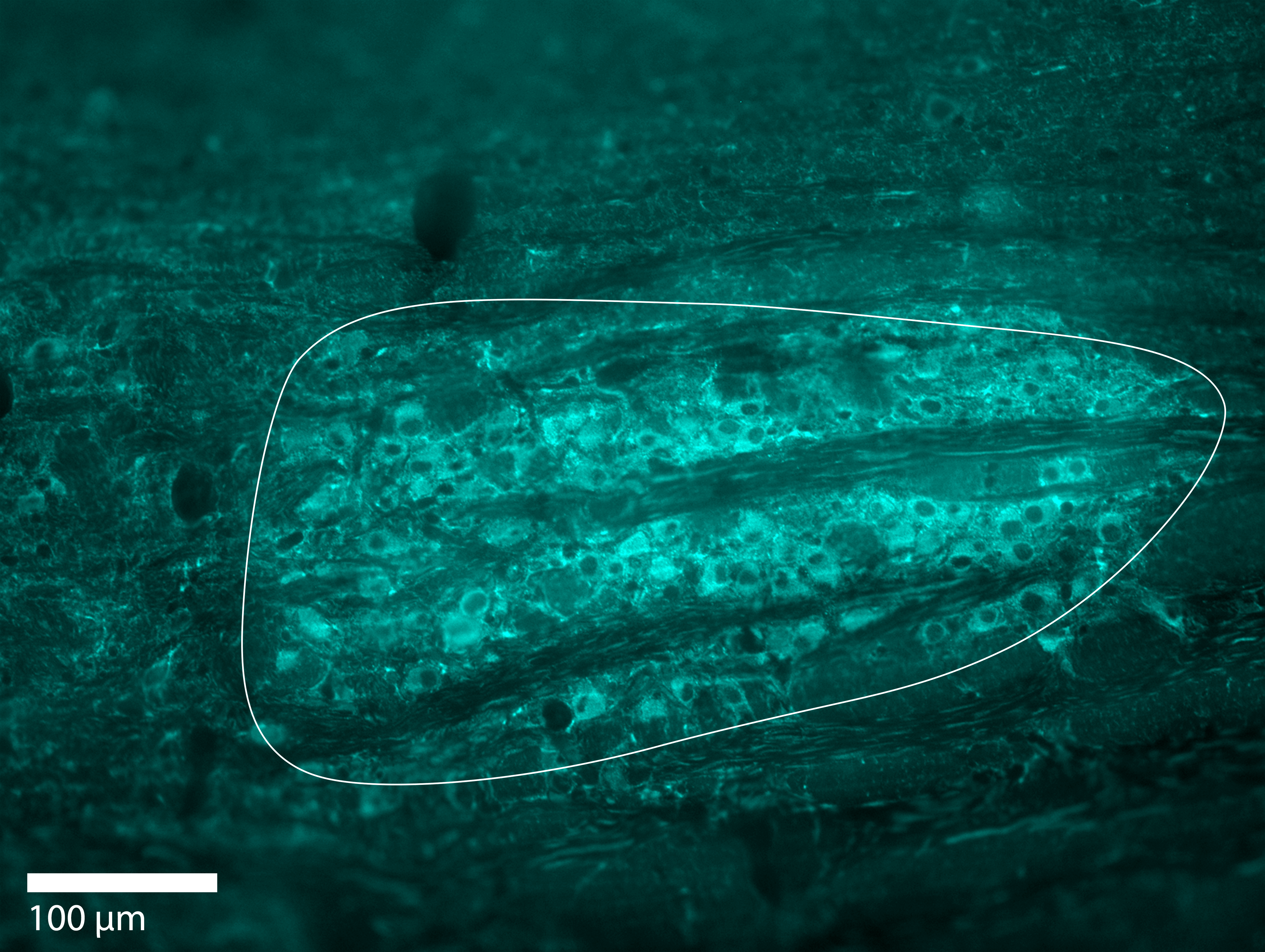

Supplement: Supplementary file 4 [file Image_2.tif]

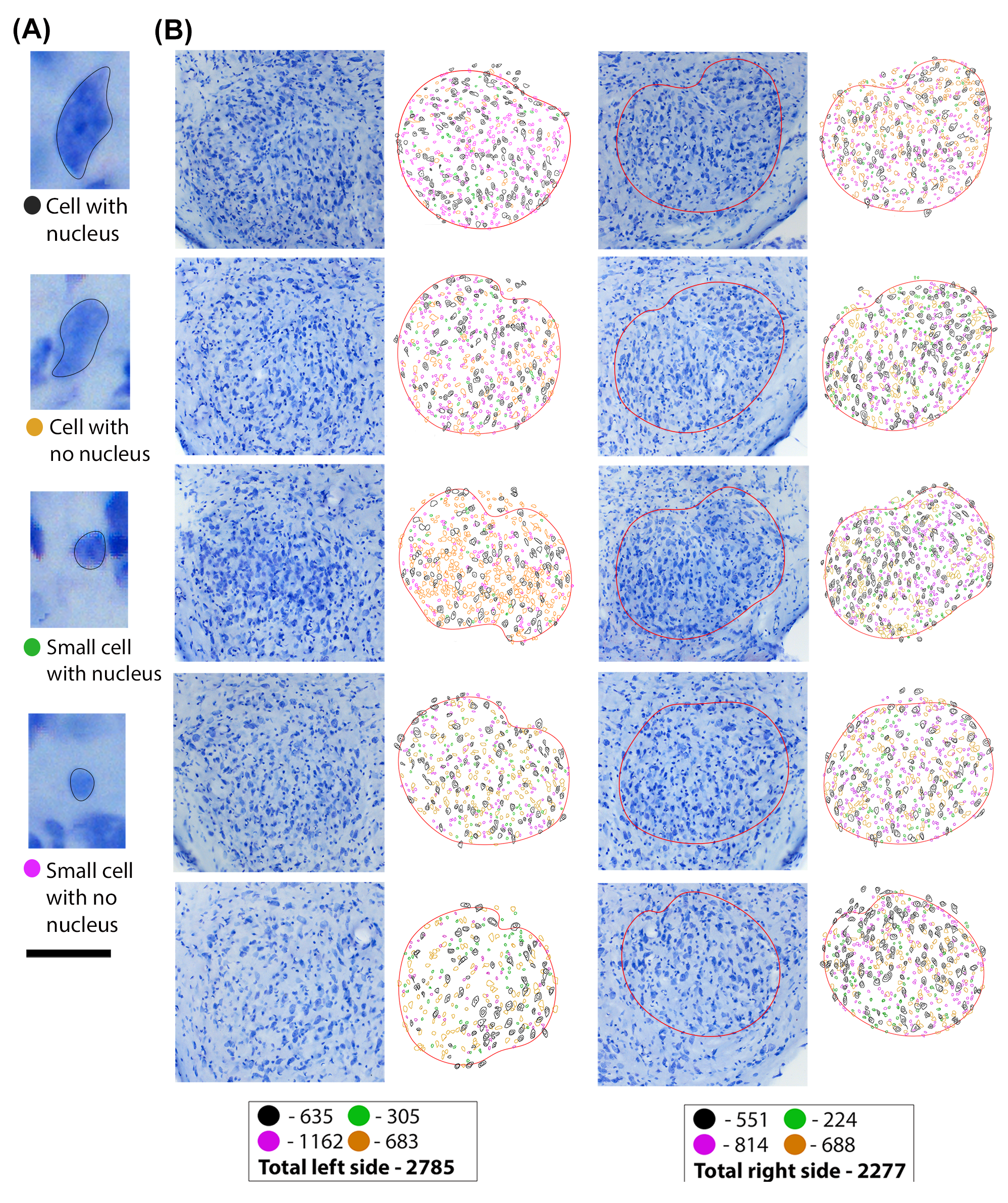

Supplement: Supplementary file 5 [file Image_3.tif]

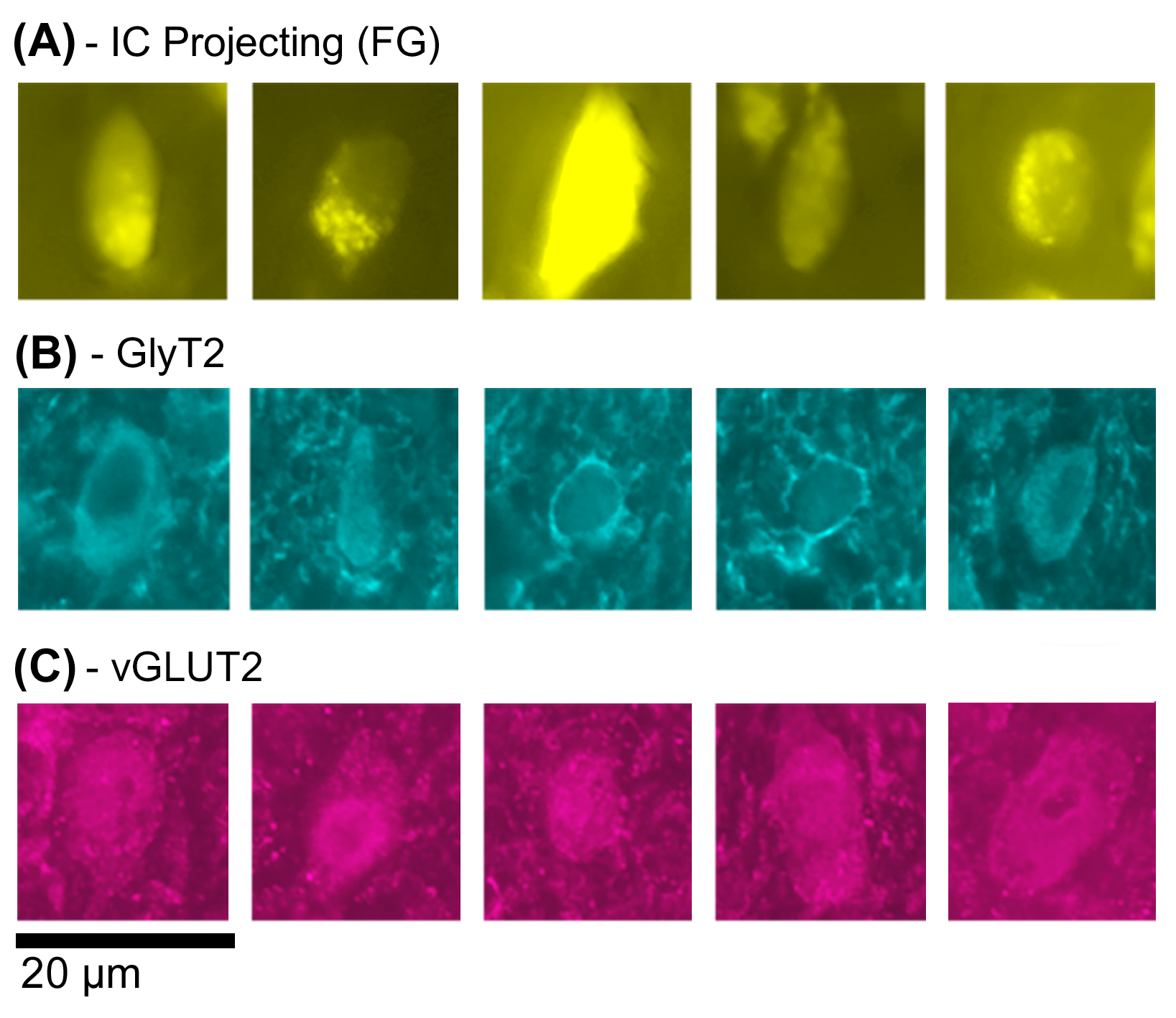

Supplement: Supplementary file 6 [file Image_4.tif]

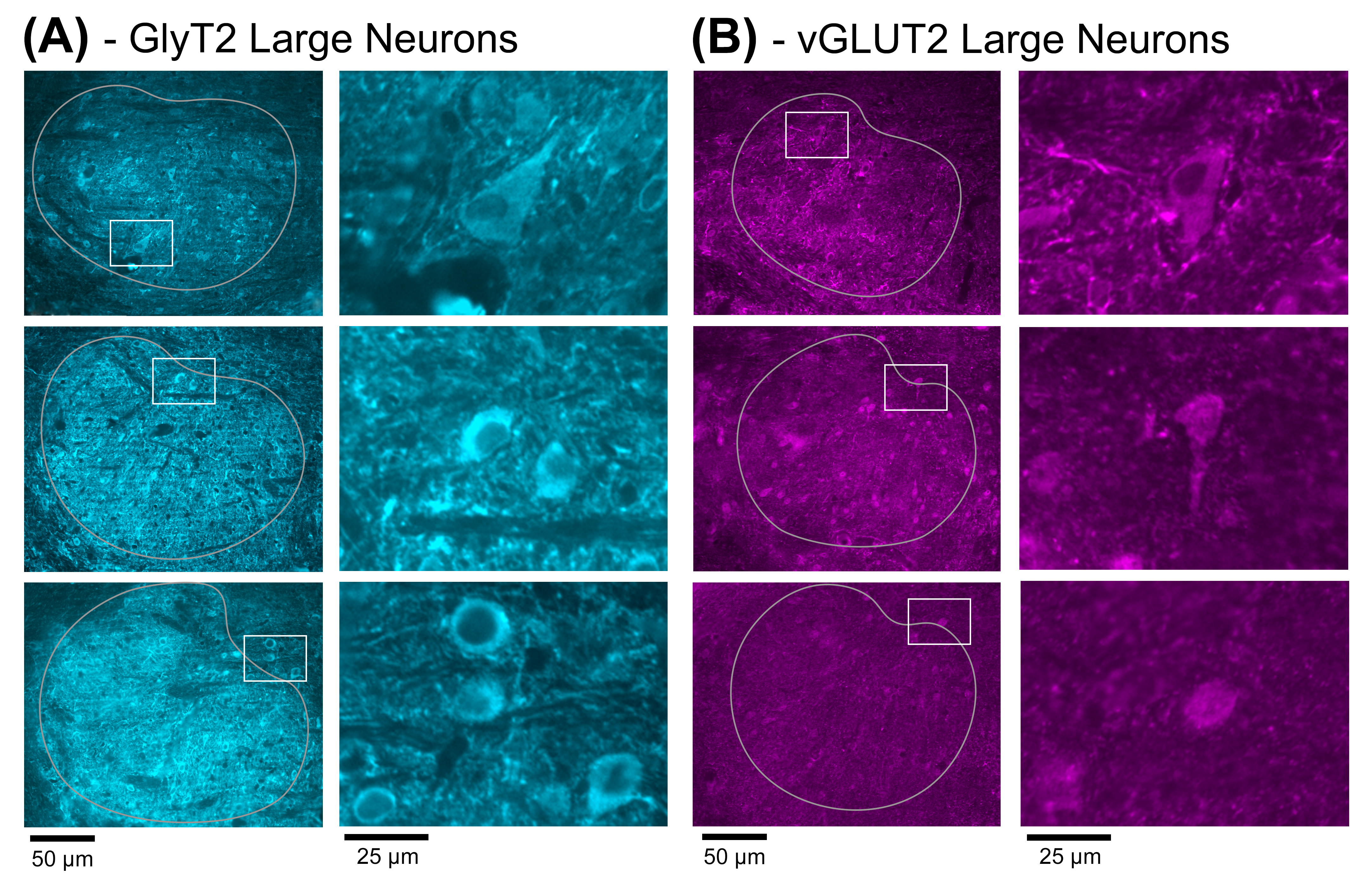

Supplement: Supplementary file 7 [file Image_5.tif]

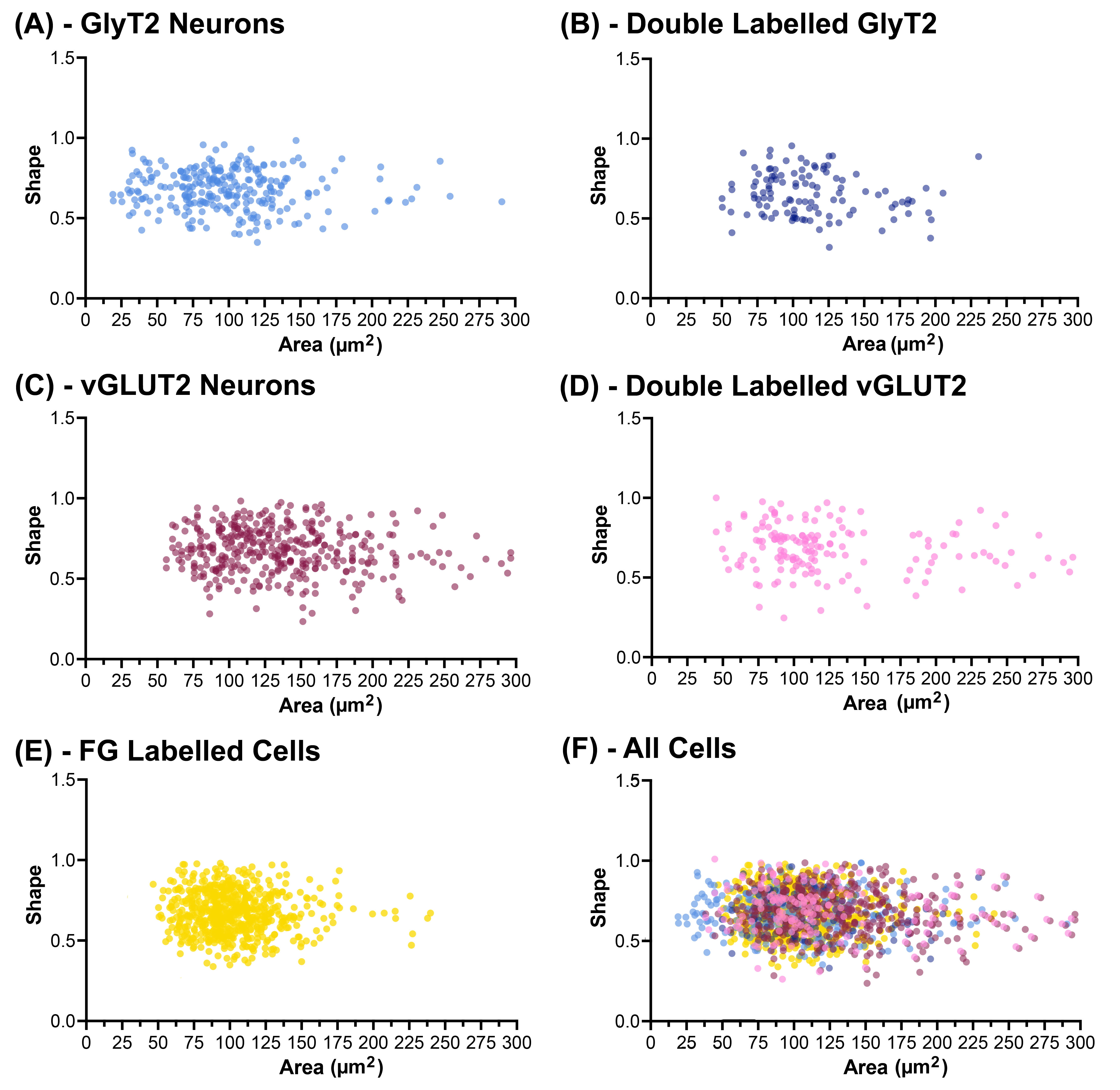

Supplement: Supplementary file 8 [file Image_6.tif]

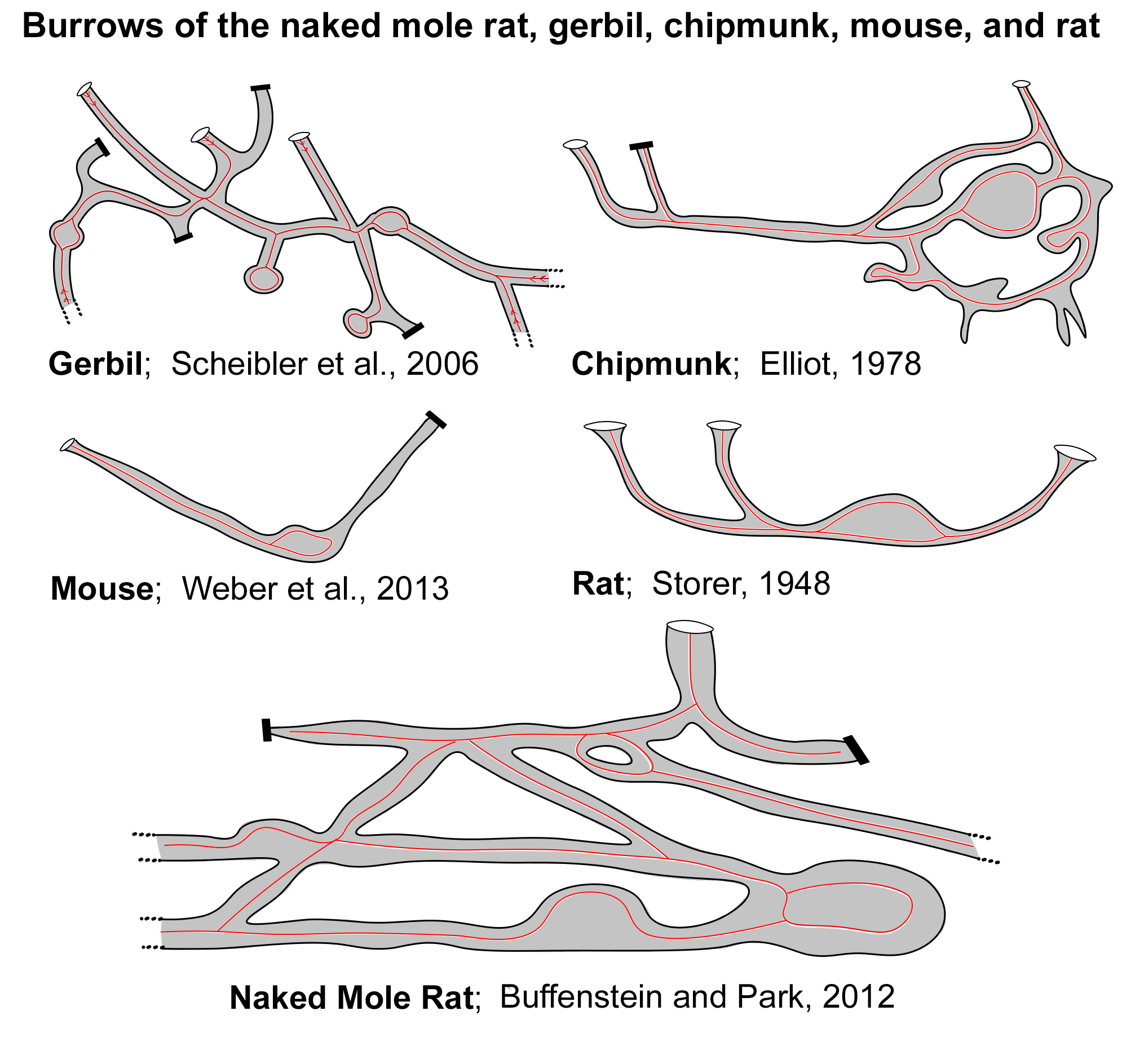

Supplement: Supplementary file 9 [file Image_7.tif]

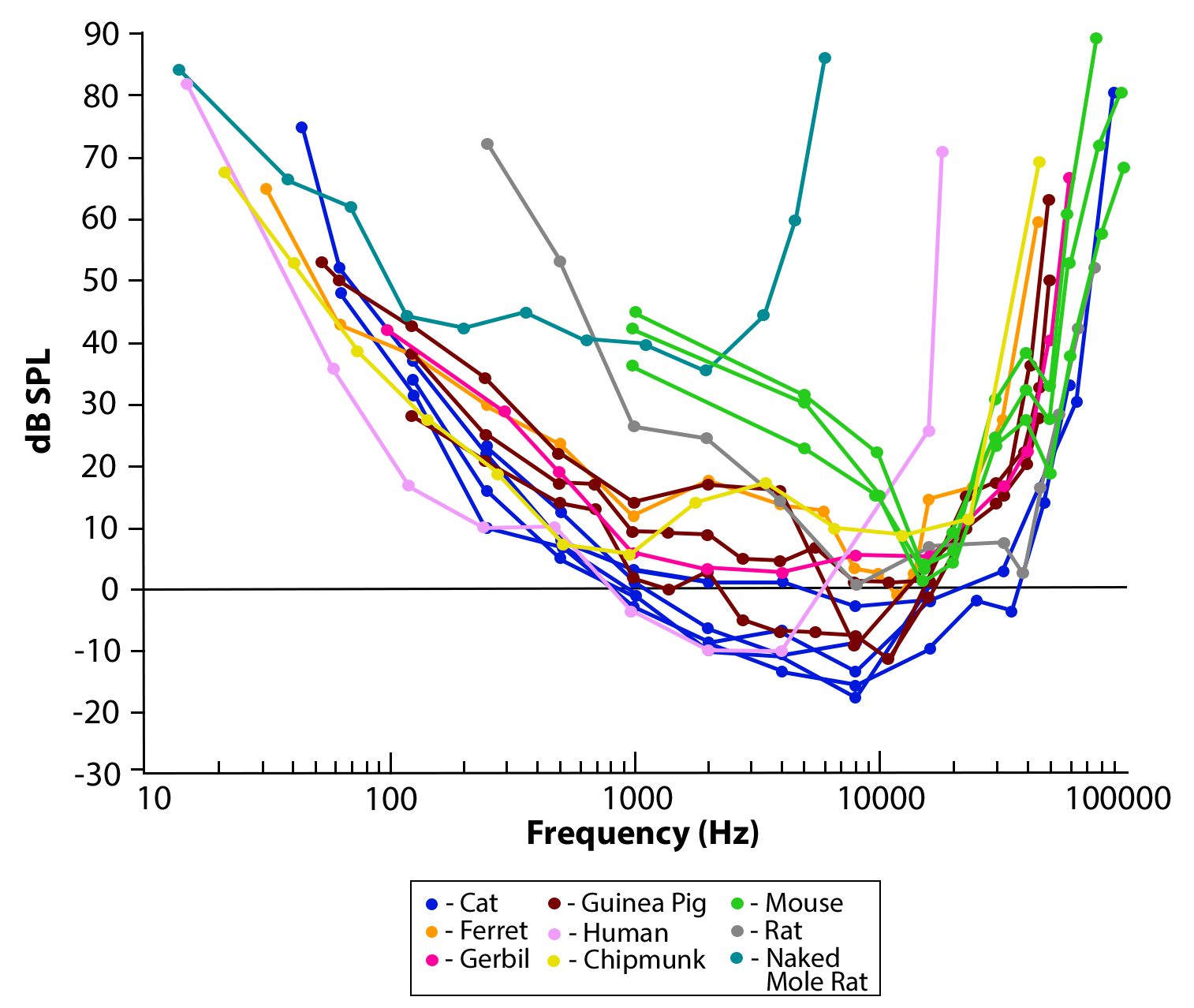

Supplement: Supplementary file 10 [file Image_8.tif]
